# Aloperine Suppresses Cancer Progression by Interacting with VPS4A to Inhibit Autophagosome‐lysosome Fusion in NSCLC

**DOI:** 10.1002/advs.202308307

**Published:** 2024-06-21

**Authors:** Weina Guo, Haifeng Zhou, Jingbo Wang, Junjie Lu, Yalan Dong, Zhenyu Kang, Xiaoyuan Qiu, Xiaohu Ouyang, Qianyun Chen, Junyi Li, Xiang Cheng, Keye Du, Mingyue Li, Zhihao Lin, Min Jin, Lei Zhang, Alexey Sarapultsev, Kuangyu Shi, Fangfei Li, Ge Zhang, Kongming Wu, Yueguang Rong, Vigo Heissmeyer, Yue Liu, Yunlun Li, Kun Huang, Shanshan Luo, Desheng Hu

**Affiliations:** ^1^ Department of Integrated Traditional Chinese and Western Medicine Union Hospital, Tongji Medical College Huazhong University of Science and Technology Wuhan 430000 China; ^2^ Department of Laboratory Medicine Wuhan Children's Hospital of Tongji Medical College Huazhong University of Science and Technology Wuhan 430000 China; ^3^ Xiangyang Central Hospital Affiliated Hospital of Hubei University of Arts and Science Xiangyang 441000 China; ^4^ Hubei Key Laboratory of Biological Targeted Therapy Union Hospital, Tongji Medical College Huazhong University of Science and Technology Wuhan 430022 China; ^5^ Department of Neurosurgery Union Hospital of Tongji Medical College Huazhong University of Science and Technology Wuhan 430000 China; ^6^ Department of Gastroenterology Zhongda Hospital, Southeast University Nanjing 210000 China; ^7^ Institute of Neuroscience, School of Medicine Xiamen University Xiamen 361000 China; ^8^ Cancer Center Union Hospital Tongji Medical College Huazhong University of Science and Technology Wuhan 430000 China; ^9^ Affiliated Hospital of Shandong University of Traditional Chinese Medicine Jinan 250014 China; ^10^ School of Medical Biology South Ural State University Chelyabinsk 454087 Russia; ^11^ Department of Nuclear Medicine University of Bern Bern 3007 Switzerland; ^12^ Shum Yiu Foon Sum Bik Chuen Memorial Centre for Cancer and Inflammation Research School of Chinese Medicine Hong Kong Baptist University Hong Kong SAR 999077 China; ^13^ Institute of Integrated Bioinfomedicine and Translational Science School of Chinese Medicine Hong Kong Baptist University Hong Kong SAR 999077 China; ^14^ Department of Oncology Tongji Hospital of Tongji Medical College Huazhong University of Science and Technology Wuhan 430000 China; ^15^ School of Basic Medicine of Tongji Medical College Huazhong University of Science and Technology Wuhan 430000 China; ^16^ Institute for Immunology Biomedical Center Ludwig‐Maximilians‐Universität München 82152 Planegg‐Martinsried Germany; ^17^ Cardiovascular Disease Center Xiyuan hospital of China academy of Chinese medical Sciences Beijing 100102 China; ^18^ Innovation Research Institute of Traditional Chinese Medicine Shandong University of Traditional Chinese Medicine Jinan 250355 China; ^19^ School of Pharmacy of Tongji Medical College Huazhong University of Science and Technology Wuhan 430030 China; ^20^ Institute of Hematology, Union Hospital Tongji Medical College Huazhong University of Science and Technology Wuhan 430000 China; ^21^ Hubei Key Laboratory of Biological Targeted Therapy China‐Russia Medical Research Center for Stress Immunology Union Hospital Tongji Medical College Huazhong University of Science and Technology Wuhan 430000 China

**Keywords:** apoptosis, autophagy inhibition, non‐small cell lung cancer, sequestosome‐1, VPS4A

## Abstract

Aloperine (ALO), a quinolizidine‐type alkaloid isolated from a natural Chinese herb, has shown promising antitumor effects. Nevertheless, its common mechanism of action and specific target remain elusive. Here, it is demonstrated that ALO inhibits the proliferation and migration of non‐small cell lung cancer cell lines in vitro and the tumor development in several mouse tumor models in vivo. Mechanistically, ALO inhibits the fusion of autophagosomes with lysosomes and the autophagic flux, leading to the accumulation of sequestosome‐1 (SQSTM1) and production of reactive oxygen species (ROS), thereby inducing tumor cell apoptosis and preventing tumor growth. Knockdown of SQSTM1 in cells inhibits ROS production and reverses ALO‐induced cell apoptosis. Furthermore, VPS4A is identified as a direct target of ALO, and the amino acids F153 and D263 of VPS4A are confirmed as the binding sites for ALO. Knockout of VPS4A in H1299 cells demonstrates a similar biological effect as ALO treatment. Additionally, ALO enhances the efficacy of the anti‐PD‐L1/TGF‐β bispecific antibody in inhibiting LLC‐derived subcutaneous tumor models. Thus, ALO is first identified as a novel late‐stage autophagy inhibitor that triggers tumor cell death by targeting VPS4A.

## Graphical abstract

Aloperine (ALO) interacts with the F153 and D263 amino acids of VPS4A, resulting in the formation of unsealed autophagosomes, thereby inhibiting the fusion of autophagosomes and lysosomes. Furthermore, ALO‐mediated blockage of autophagic flux induces the accumulation of sequestosome (SQSTM1), which promotes excessive reactive oxygen species production (ROS), ultimately leading to apoptosis of non‐small cell lung cancer cells.

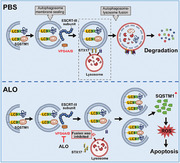

## 1 Introduction

Lung cancer is known as the most common oncological disease, accounting for 11.6% and 18.4% of global cancer morbidity and mortality, respectively.^[^ ^1^ ^]^ According to histological classification, ≈85% of lung cancer patients are non‐small cell lung cancer (NSCLC).^[^ ^2^ ^]^ Despite recent therapeutic advancements in NSCLC, progressing from chemotherapy to tyrosine kinase inhibitors (TKIs) and immune checkpoint blocker treatments,^[^ ^2^ ^]^ some limitations still remain. For example, one major challenge is the failure to maintain oncotherapy responses due to the emergence of resistant secondary clones. Therefore, there is an urgent need to identify novel anti‐cancer agents complementary to chemotherapy, TKIs, or immunotherapy for effectively treating NSCLC patients.

Macroautophagy/autophagy is an evolutionarily conserved physiological process triggered by cellular stress or nutrient deprivation to meet metabolic and energy requirements of cells through the recycling of intracellular compounds.^[^ ^1^ ^]^ In general, cellular proteins and organelles are sequestered in autophagosomes and subsequently fused with lysosomes for further degradation and recycling.^[^ ^2^ ^]^ In fact, autophagy plays a complex role in tumorigenesis, either inhibiting or promoting tumor progression. On the one hand, liver‐specific deletion of ATG5 or ATG7 was reported to induce benign hepatomas,^[^ ^3^ ^]^ suggesting a role for autophagy in tumor suppression. On the other hand, autophagy can provide nutrients for tumor cells, promoting tumor progression and aiding in evading immune surveillance and developing drug resistance.^[^ ^1^ ^]^ Indeed, clinical trials targeting autophagy in lung cancer have made notable progress in recent years. For instance, a phase Ib/II clinical trial revealed that hydroxychloroquine, a common autophagy inhibitor, could reverse chemotherapy resistance in advanced lung cancer.^[^ ^4^ ^]^ Nevertheless, the low efficiency of most autophagy inhibitors restricts their clinical application. Thus, the development of novel autophagy inhibitors with better efficacy and specificity holds significant clinical importance.

The endosomal sorting complexes required for transports (ESCRTs), consisting of ESCRT‐0 to ‐III and VPS4 subunits, have proven to be crucial for membrane fission and remodeling, thereby participating in various processes including membrane repair and autophagy.^[^ ^5^ ^]^ The ESCRT Enzymes VPS4, an AAA+ ATPase enzyme comprising VPS4A and VPS4B, is required for the disassembly of ESCRT‐III,^[^ ^6^ ^]^ which is essential for the fusion of autophagosomes with lysosomes.^[^ ^7^ ^]^ VPS4A and VPS4B are homologs of VPS4 gene, and it has been shown that low levels or absence of VPS4B renders cells more dependent on VPS4A.^[^ ^8^ ^]^

Reactive oxygen species (ROS) play dual roles in cancer. The common consensus suggests that ROS promote cancer progression, and elevated ROS is a hallmark of cancer.^[^ ^9^ ^]^ However, beyond a certain threshold, ROS can also induce cancer cell apoptosis and have been proposed as a common mediator of cell death. It is noteworthy that ROS have been reported to participate in the crosstalk between autophagy and apoptosis in cancer cells. When the autophagy process is inhibited, oxidative stress induces elevated ROS levels, ultimately leading to apoptosis.^[^ ^10^ ^]^ Despite autophagy and apoptosis proceeding through independent mechanisms, accumulating evidence indicates the existence of interplay between the two pathways. Inhibition of one pathway may enhance or inhibit the other pathway.^[^ ^11^ ^]^ For instance, treatment of deprived cells with autophagy inhibitors accelerates apoptotic cell death.^[^ ^12^ ^]^

Aloperine (ALO), a quinolizidine‐type alkaloid isolated from the seeds and leaves of *Sophora alopecuroides L*., has been reported to exhibit potent antitumor effects on various types of cancer, including colon cancer cells,^[^ ^13^ ^]^ multiple myeloma cells,^[^ ^14^ ^]^ and thyroid cancer cells.^[^ ^15^ ^]^ The identified mechanisms mainly involve inducing apoptosis, suppressing invasion and inhibiting autophagy, suggesting critical engagement of these processes. Nevertheless, the molecular mechanism underlying ALO's antitumor effects on NSCLC and its specific molecular target in NSCLC cells remain largely unknown.

In this study, we revealed the antitumor effect of ALO on NSCLC both in vitro and in vivo. The results showed that ALO‐induced cytotoxicity was due to its capacity to inhibit autophagy. Our findings demonstrate that ALO directly binds and inhibits VPS4A, acting as a “molecular brake” that hampers the fusion of autophagosomes with lysosomes, thereby inhibiting the late stages of autophagy. Furthermore, ALO induces increased SQSTM1 level, leading to progressive ROS accumulation, ultimately resulting in cell death. Knockdown of *SQSTM1* abolished ALO‐induced ROS accumulation and cytotoxicity. These findings identify ALO as a novel late‐stage autophagy inhibitor that efficiently prevents NSCLC tumor growth by targeting VPS4A.

## 2 Results

### 2.1 ALO Inhibits Proliferation and Migration of NSCLC Cell Lines

**Figure 1:**
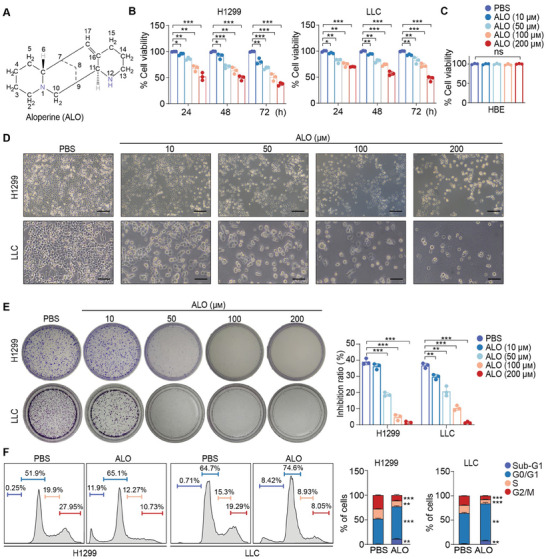
ALO inhibits cell proliferation in NSCLC cells. A) The chemical structure of ALO with a molecular weight of 232.365 g mol^−1^. B) Cell viability of H1299 and LLC cells treated with ALO (0–200 µм) for indicated time periods was determined by the CCK‐8 assay (n = 3). C) Cell viability of normal human bronchial epithelioid (HBE) cells treated with the ALO (0–200 µм) for 24 h was determined by the CCK‐8 assay. D) Microscopy images of H1299 and LLC cells treated with the ALO (0–200 µм) for 24 h. Scale bar: 100 µm. E) The macrographs and quantitative analyses of clone formation of H1299 and LLC cells (n = 3). F) Representative results of cell cycle analysis and quantitative analyses after treatment with ALO (200 µм) for 24 h (n = 3). Data in B–E) are presented as mean ± SD, and *p* values were calculated using one‐way ANOVA. Data in F) are presented as mean ± SD, and *p* values were determined by two‐tailed unpaired Student's t‐test. ns, not significant; *p < 0.05, **p < 0.01, ***p < 0.001.

To evaluate the cytotoxicity and inhibitory effects of ALO on NSCLC cells, human NSCLC cell line H1299 and mouse NSCLC cell line Lewis lung carcinoma (LLC) cells were treated with ALO (**Figure** **1**A). First, H1299 and LLC cell lines were treated with different concentrations of ALO for diverse time periods, and cell viability was subsequently analyzed by cell counting kit‐8 (CCK‐8). The results demonstrated that ALO inhibited the growth of H1299 and LLC cells in a time‐ and dose‐dependent manner (Figure 1B). Interestingly, ALO‐mediated growth inhibition was not observed in normal human bronchial epithelioid (HBE) cells (Figure 1C), indicating selective cytotoxicity against NSCLC cells rather than HBE cells. Subsequently, cell proliferation was assessed following ALO treatment. Consistently, ALO‐mediated growth inhibition of NSCLC cells was further validated through microscopy images (Figure 1D). Second, ALO‐mediated anti‐proliferative effect was determined using a colony formation assay. The results showed that ALO treatment significantly suppressed the colony formation in both H1299 and LLC cells in a dose‐dependent manner (Figure 1E), indicating a general inhibition of cell proliferation by ALO. Third, to further confirm the impact of ALO on cell proliferation, the cell cycle was examined following ALO treatment. The results indicated that ALO arrested the cell cycle in the G0/G1 phase in both H1299 and LLC cells, leading to a significant reduction in cells in the S and G2/M phases (Figure 1F). Intriguingly, ALO also increased apoptotic sub‐G1 population in both H1299 and LLC cells. Taken together, these results demonstrate that ALO potently reduces cell proliferation and viability by inducing sub‐G1 and G0/G1‐phase arrest.

Migration and invasion are critical processes in tumor metastasis. Consequently, the effects of ALO on the migration of H1299 and LLC cells were initially investigated. To this end, a wound healing assay was performed. The results demonstrated that treating H1299 and LLC cells with ALO significantly reduced cellular migration as compared with non‐treated control group (Figure S1A,B, Supporting Information). Consistently, Transwell assays confirmed the inhibitory effects of ALO on the migration and invasion of H1299 and LLC cells (Figure S1C,D, Supporting Information). In addition, epithelial‐mesenchymal transition (EMT) is a cellular program crucial for cancer metastasis. Therefore, the effects of ALO on EMT in H1299 and LLC cells were also assessed following ALO treatment. The data showed that ALO downregulated mesenchymal markers such as cadherin‐2, matrix metalloproteinase‐9, vimentin, and SNAI2, while upregulating the epithelial marker cadherin‐1, as confirmed at both mRNA and protein levels (Figure S1E,F, Supporting Information), indicating that ALO inhibits cell migration and metastasis of NSCLC cell lines in vitro by suppressing the EMT process.

### 2.2 ALO Exerts Antitumor Efficacy In Vivo

**Figure 2:**
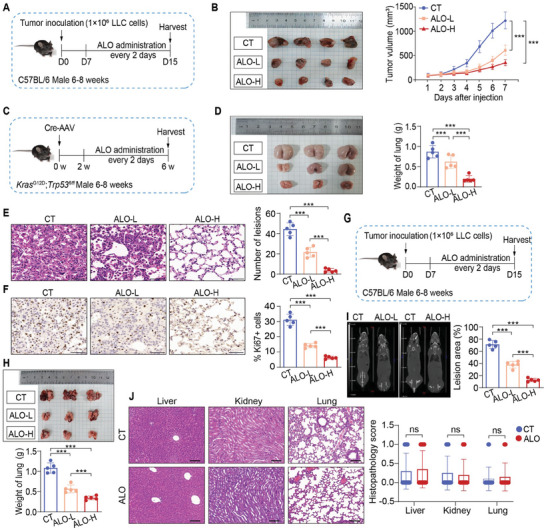
ALO exerts antitumor efficacy in vivo. A) C57BL/6J mice with LLC‐derived subcutaneous tumors were administered with low‐dose ALO (10 mg kg^−1^, ALO‐L) or high‐dose ALO (50 mg kg^−1^, ALO‐H) or PBS (Control, CT) every 2 days (n = 5). B) Images of LLC‐derived subcutaneous tumors and tumor growth curves revealed a significant inhibitory effect of ALO on the growth of the LLC‐derived subcutaneous tumors. C) Schematic diagram of injection protocol in *Kras* ^G12D^; *Trp53^fl/fl^* mice (n = 5). D) Images and weights of tumors in *Kras* ^G12D^; *Trp53^−/−^* mice. E) H&E staining and histopathological analysis of lungs collected from the *Kras* ^G12D^; *Trp53^−/−^* mice (n = 5). Scale bar: 50 µm. F) Immunohistochemistry analysis of Ki67 in tumor sections from the *Kras* ^G12D^; *Trp53^−/−^* mice (n = 5). Scale bar: 50 µm. G) Schematic diagram of injection protocol in mice with LLC‐derived lung metastasis tumors (n = 5). H) Images and weights of tumors in mice with LLC‐derived lung metastasis tumors (n = 5). I) Computed Tomography analysis of LLC‐derived lung metastasis tumors (n = 5). J) H&E staining and histopathological analysis of liver, kidney and lung collected from the control (CT) and high‐dose ALO‐treated group (n = 5). Scale bar: 100 µm. Data in B–I) are presented as mean ± SD, and *p* values were calculated using one‐way ANOVA. Data in J) are presented as mean ± SD, and *p* values were determined by two‐tailed unpaired Student's t‐test. ns, not significant; *p < 0.05, **p < 0.01, ***p < 0.001.

To evaluate whether ALO exhibits therapeutic effects on NSCLC tumor growth in vivo, H1299 and LLC cells were subcutaneously injected into BALB/c nude mice and C57BL/6 mice, respectively, to establish a subcutaneous tumor model. Seven days after infection, mice were randomly divided into three groups: control group (CT), the low‐dose ALO group (ALO‐L) and the high‐dose ALO group (ALO‐H), and administered with intraperitoneal injection of PBS or ALO every two days for 8 days (**Figure** **2**A; Figure S2A, Supporting Information). Throughout the ALO administration period, no significant differences in body weight were observed between the CT and ALO‐treated groups (data not shown). Notably, the LLC‐derived subcutaneous tumor growth was significantly suppressed by both low and high dose of ALO, as evidenced by the decreased tumor volumes in ALO‐treated group compared with the CT group (Figure 2B). Likewise, reduced volumes of H1299‐derived subcutaneous tumor were observed in ALO‐treated group compared with the CT group (Figure S2B,C, Supporting Information).

Additionally, the effects of ALO on the progression of lung adenocarcinoma in the cre‐recombinase *Kras* ^G12D^; *Trp53^fl/fl^* mutation mouse model were investigated. *Kras* ^G12D^; *Trp53^fl/fl^* mice were randomly divided into three groups (CT, ALO‐L and ALO‐H) and administered intraperitoneal injections of PBS or ALO every two days for 4 weeks (Figure 2C). The results showed that mice tolerated the administered dose of ALO well without significant changes in body weight (data not shown). In addition, the tumors were significantly inhibited, as evidenced by decreased tumor weight in mice with ALO administration compared with the CT group (Figure 2D). Hematoxylin and eosin (H&E) staining revealed a significant reduction in the degree of adenomatous hyperplasia in the ALO‐L and ALO‐H groups compared with the CT group (Figure 2E). Besides, the level of Ki‐67 was significantly decreased after ALO treatment, as demonstrated by immunohistochemistry analysis of the tumor tissues (Figure 2F), suggesting that ALO inhibited the growth of lung adenocarcinoma. Consistently, LLC cells were intravenously injected into C57BL/6J mice to establish a lung metastasis tumor model. Mice were randomly divided into three groups (CT, ALO‐L and ALO‐H) and administered with PBS or ALO every two days for 8 days (Figure 2G). Notably, ALO treatment significantly suppressed lung tumor growth compared with the CT group (Figure 2H), which was further confirmed by the computed tomography of lung metastasis tumor (Figure 2I).

Subsequently, the in vivo side effects of ALO were assessed simultaneously with the above in vivo experiments. First, various organs were harvested, sectioned and stained with H&E. Histopathological analysis revealed no histological differences in the liver, kidney, or lung between the CT and ALO‐treated groups (Figure 2J). In addition, no obvious differences were detected between the CT and ALO‐treated groups in terms of serum levels of alanine transaminase, aspartate aminotransferase, blood urea nitrogen and creatinine, which are biochemical indices for liver and kidney function (Figure S2D, Supporting Information). Additionally, no significant differences were observed in the morphology and weight of the spleens and thymuses, nor in the total number of cells in the bone marrows (BMs), spleens, mesenteric lymph nodes (MLNs), and thymuses of ALO‐treated mice compared with the CT group (Figure S2E,F, Supporting Information). Consistently, leukocytes were isolated from BM, spleen, thymus, MLN, peripheral blood and tumors, and the percentage of myeloid cells (neutrophils, macrophages, dendritic cells, and monocytes) and lymphoid cells (NK cells, B cells, and T cell subsets) in BM, spleen, MLN, and peripheral blood mononuclear cells (PBMCs), as well as T cell subsets in the thymus, were detected by flow cytometry. The results showed no significant differences in the proportions of detected immune cell subsets between the CT group and the ALO group (Figures S2G–K and S3A,B, Supporting Information). Taken together, these data demonstrate that ALO has the therapeutic potential to inhibit NSCLC growth without obvious side effects.

### 2.3 Identification of ALO as an Autophagy Modulator

**Figure 3:**
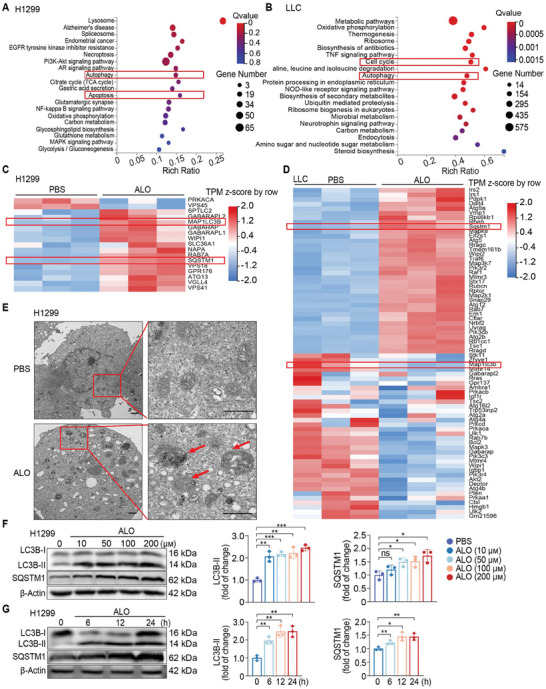
Identification of ALO as an autophagy modulator. A,B) The Kyoto Encyclopedia of Genes and Genomes bubble map of differentially enriched genes in H1299 A) and LLC cells B) treated with the PBS or ALO (200 µм) for 12 h (n = 3). C,D) Heatmaps of differentially expressed genes in the autophagy pathways of ALO‐treated and untreated H1299 C) and LLC cells D). E) Transmission electron micrographs of H1299 cells treated with PBS or ALO (200 µм) for 24 h. The right pictures are the enlarged representations of the boxed regions of the left pictures. Scale bar: 1 µm. F) Immunoblotting assays were performed to assess LC3B‐II and SQSTM1 levels in H1299 cells treated with ALO (0–200 µм) for 24 h and quantified by gray scale analysis (n = 3). G) Immunoblotting assays were performed to assess LC3B‐II and SQSTM1 levels in H1299 cells treated with ALO (200 µм) for indicated time periods and quantified by gray scale analysis (n = 3). β‐Actin was used as a loading control. Data in F,G) are presented as mean ± SD, and *p* values were calculated using one‐way ANOVA. ns, not significant; *p < 0.05, **p < 0.01, ***p < 0.001.

ALO exhibits strong anti‐NSCLC tumor effects both in vitro and in vivo, yet the underlying molecular mechanism requires elucidation. Transcriptome analysis was performed to identify the differentially expressed genes between the ALO‐treated and untreated NSCLC cells. Kyoto Encyclopedia of Genes and Genomes (KEGG) pathway enrichment analysis revealed enrichment of the cell cycle pathway, with ALO decreasing the expression of cell cycle‐related genes including CCND1 and CDK4 in NSCLC cells (Figure S4A,B, Supporting Information). It is noteworthy that the autophagy pathway and related genes were enriched after ALO treatment in H1299 and LLC cells (**Figure** **3**A–D). Considering that autophagy plays a pivotal role in cancer cell survival under stress,^[^ ^16^ ^]^ we speculated that modulation of autophagy might be a key determinant for ALO‐induced cellular effects. Furthermore, cytoplasmic vacuolization was observed in H1299 and LLC cells after treatment with ALO for 12 h (Figure S4C, Supporting Information), suggesting that this compound might regulate autophagy. Notably, an increased number of autophagic vesicles (autolysosomes and autophagosomes) were observed in ALO‐treated cells using transmission electron microscopy (Figure 3E; Figure S4D, Supporting Information).

The levels of microtubule‐associated protein 1 light chain 3 (LC3) and SQSTM1 are widely used as bio‐markers of autophagy. LC3 usually exhibits a molecular conversion from cytoplasmic LC3‐I into its lipidated counterpart LC3‐II, which is recruited to phagophores and stays on the inner membrane of the autophagosome until degraded in the autolysosome. Consequently, the level of LC3‐II is widely utilized to monitor autophagosome levels.^[^ ^17^ ^]^ Interestingly, immunoblotting assays revealed that ALO increased the ratio of LC3B‐II/β‐Actin and the level of sequestosome‐1 (SQSTM1) in a dose‐ and time‐dependent manner both in H1299 and LLC cells (Figure 3F,G; Figure S4E,F, Supporting Information), suggesting that ALO treatment enhanced the accumulation of autophagosomes.

### 2.4 ALO Functions as a Late‐stage Autophagy Inhibitor

**Figure 4:**
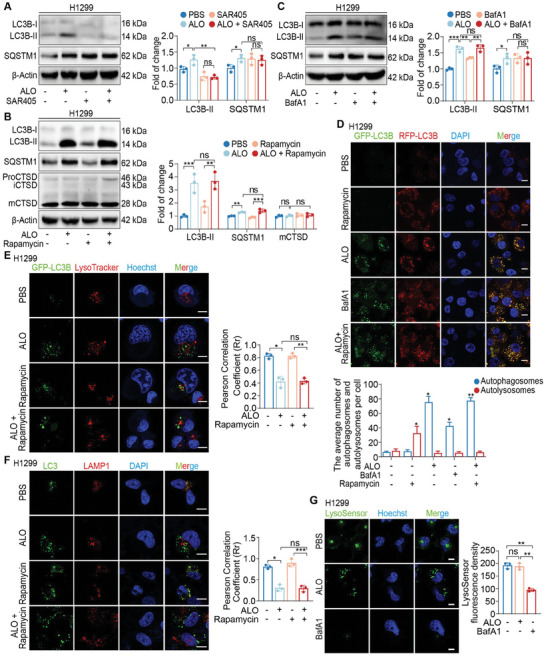
ALO functions as a late‐stage autophagy inhibitor. A) Immunoblotting assays were performed to assess LC3B‐II and SQSTM1 levels in H1299 cells treated with PBS or ALO (200 µм) in the absence or presence of SAR405 (10 µм) for 2 h and quantified by gray scale analysis (n = 3). B) Immunoblotting assays were performed to assess LC3B‐II, SQSTM1 and procathepsin D (pro‐CTSD), preprocathepsin D (pre‐CTSD) and mature CTSD (mCTSD) in H1299 cells treated with PBS or ALO (200 µм) in the absence or presence of rapamycin (250 nм, 24 h) for 2 h and quantified by gray scale analysis (n = 3). C) Immunoblotting assays were performed to assess LC3B‐II and SQSTM1 levels in H1299 cells treated with PBS or ALO (200 µм) in the absence or presence of 1 µм Bafilomycin A1 (BafA1) for 2 h and quantified by gray scale analysis (n = 3). D) Fluorescence images of H1299 cells transfected with RFP‐GFP‐LC3B reporter and analysis of the number of autophagosomes and autolysosomes. Cells were treated with PBS or ALO (200 µм) in complete medium for 2 h. 1 µм BafA1‐treated cells were used as positive controls (n = 3). Scale bar: 10 µm. E) Fluorescence images and analysis of the colocalization of GFP‐LC3B and LysoTracker Red in H1299 cells cultured in complete medium in the absence or presence of ALO (200 µм) for 2 h (n = 3). Scale bar: 10 µm. F) Immunofluorescence images and analysis of the colocalization of LC3 (green) and LAMP1 (red) in H1299 cells treated with PBS or ALO (200 µм) for 2 h (n = 3). Scale bar: 10 µm. G) H1299 cells were treated with PBS or ALO (200 µм) for 2 h. Representative images of H1299 cells stained with LysoSensor Green and quantification of fluorescence intensity (n = 3). 1 µм BafA1‐treated cells were used as positive controls. Scale bar: 10 µm. Data in A–G) are presented as mean ± SD, and *p* values were calculated using one‐way ANOVA. ns, not significant; *p < 0.05, **p < 0.01, ***p < 0.001.

It has been proved that ALO promotes the accumulation of autophagosomes. Nevertheless, whether ALO induces the production or inhibits the degradation of autophagosomes requires further analysis. It is hypothesized that the increased LC3B‐II could be attributed to either increased autophagosome formation or impaired degradation of autophagosomes. To discriminate these two possibilities, NSCLC cells were treated with ALO in the presence of SAR405 or small interfering RNA (siRNA) targeting *ATG7*, which inhibits early‐stage autophagy by suppressing autophagosome formation, or bafilomycin A1 (BafA1), which inhibits late‐stage autophagy by impairing lysosomal degradation. The autophagic flux was then analyzed by immunoblotting. The results demonstrated that the effects of ALO on LC3B‐II accumulation were significantly reduced when co‐incubated with SAR405 or transfected with *ATG7* siRNA, which suppressed the upstream steps of autophagy (**Figure** **4**A; Figure S5A–C, Supporting Information). Notably, co‐incubation of cells with ALO evoked a more substantial accumulation of LC3B‐II compared with the autophagy inducer rapamycin or Tat‐beclin 1 alone (Figure 4B; Figure S5D,E, Supporting Information), confirming that ALO inhibits the degradation of autophagosomes. Interestingly, ALO evoked a more pronounced accumulation of LC3B‐II in NSCLC cells compared with BafA1 treatment alone (Figure 4C; Figure S5F, Supporting Information). In addition, notable increases in LC3B‐II levels were observed with ALO and Tat‐beclin 1 co‐treatment compared with Bafilomycin A1 and Tat‐beclin 1 co‐treatment in H1299 and LLC cells (Figure S5E, Supporting Information). Intriguingly, no significant increases were observed in LC3B‐II levels with co‐treatment of ALO and rapamycin compared with ALO alone (Figure 4B; Figure S5D, Supporting Information), while co‐incubation of H1299 or LLC cells with ALO and the mTOR‐independent autophagy activator Tat‐beclin 1 evoked a significant accumulation of LC3B‐II compared with ALO alone (Figure S5E, Supporting Information). Consistently, the activation of the Akt/mTOR pathway decreased with ALO treatment in NSCLC cells in vitro and mouse tumor models in vivo, whereas the levels of Beclin‐1 and ATG7 increased (Figure S6, Supporting Information), indicating that ALO may promote autophagosome formation by interfering with the Akt/mTOR pathway.

SQSTM1 is a selective autophagy receptor that is recruited to phagophores and constitutively degraded during autophagy. Accordingly, SQSTM1 is commonly used to indirectly reflect autophagic flux within cells.^[^ ^18^ ^]^ The results showed that SQSTM1 levels were notably upregulated upon treatment of both H1299 and LLC cells with ALO in a dose‐ and time‐dependent manner (Figure 3F,G; Figure S4E,F, Supporting Information), suggesting that ALO may inhibit autophagic flux.

To corroborate the effects of ALO on autophagic flux, a tandem RFP‐GFP‐LC3B reporter was applied to lung cancer cell lines. Upon treatment of NSCLC cells with ALO, fluorescence microscopy was utilized to analyze the changes in fluorescence within the cells. GFP, but not RFP, loses its fluorescence in the acidic and proteolytic milieu of lysosomes. The co‐localization of GFP with RFP signals as yellow puncta indicates autophagosomes, whereas autolysosomes exhibit solely red fluorescence.^[^ ^19^ ^]^ The results showed that ALO treatment significantly augmented the number of yellow puncta in H1299 cells, with even more pronounced fluorescence observed compared with BafA1 treatment (Figure 4D; Figure S7A, Supporting Information), indicating ALO's superiority as a late‐stage autophagy inhibitor. Conversely, rapamycin‐treated cells displayed numerous red puncta with few yellow puncta (Figure 4D; Figure S7A, Supporting Information). These findings collectively confirm that ALO is a late‐stage autophagy inhibitor.

To further investigate whether ALO affects the fusion of autophagosomes with lysosomes, the colocalization of GFP‐LC3B and LysoTracker Red was assessed, which is a fluorescent dye for labeling and tracking acidic organelles such as lysosomes in live cells. The data showed that most GFP‐LC3B puncta did not colocalize with LysoTracker Red in cells co‐cultured with ALO in the presence or absence of rapamycin (Figure 4E; Figure S7B, Supporting Information), suggesting that ALO inhibits the fusion of autophagosomes with lysosomes. Consistently, the colocalization of endogenous LC3 with lysosomal associated membrane protein 1 (LAMP1), a marker for lysosomal and endosomal membranes, was assessed using immunofluorescence microscopy. ALO treatment induced a significant separation of LC3B puncta and LAMP1, which was completely different from rapamycin‐induced changes (Figure 4F; Figure S7C, Supporting Information). These findings confirm that ALO suppresses the fusion of autophagosomes with lysosomes.

Next, whether ALO also affects the pH or hydrolytic function of lysosomes was investigated, both of which are indispensable for the degradation of autophagosomes.^[^ ^20^ ^]^ To evaluate whether ALO affected lysosomal acidification, the pH‐sensitive dye Lysosensor Green was utilized. The results indicated that the fluorescence intensity of LysoSensor Green remained unchanged with ALO treatment, suggesting that ALO did not alter lysosomal pH in NSCLC cells (Figure 4G; Figure S7D, Supporting Information). It is known that Cathepsin D (CTSD) is one of the major lysosomal aspartic proteases essential in the autophagy‐lysosomal system.^[^ ^19^ ^]^ Upon treatment of H1299 and LLC cells with ALO, the activities of mature CTSD (mCTSD) were detected by immunoblotting. The results showed that ALO did not alter the levels of mCTSD in either LLC or H1299 cells under ALO treatment (Figure 4B; Figure S5D, Supporting Information), suggesting that the hydrolytic function of lysosomes is not impaired upon ALO treatment.

### 2.5 ALO‐mediated SQSTM1 Accumulation Triggers ROS Production and Cell Apoptosis

**Figure 5:**
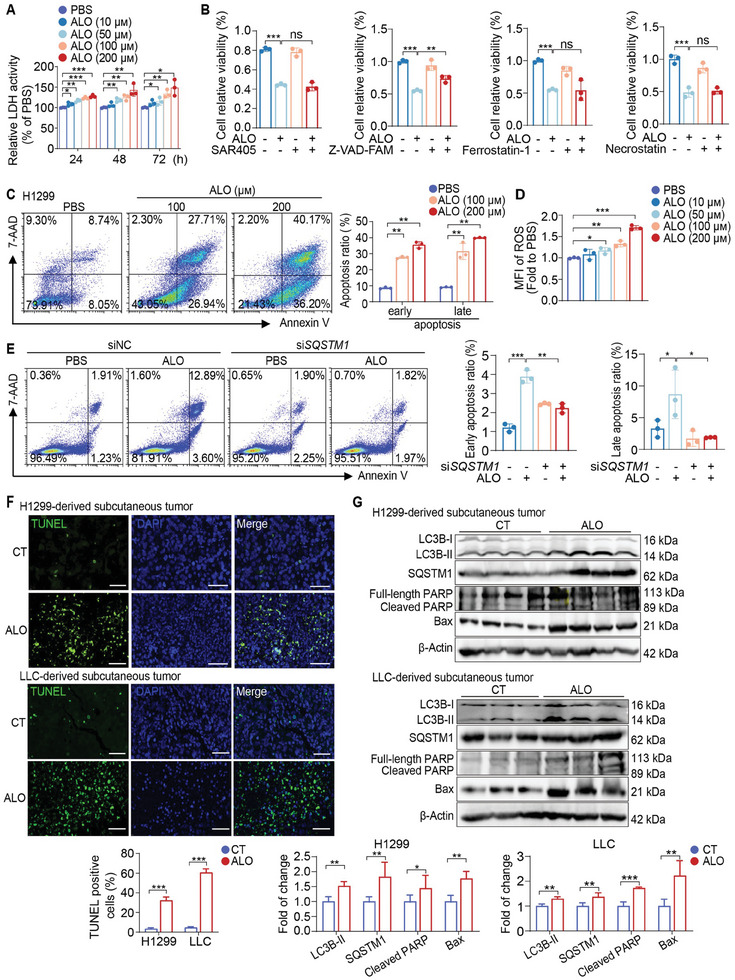
ALO‐mediated cell apoptosis is mainly induced by SQSTM1 accumulation‐mediated excessive reactive oxygen species (ROS) production. A) The cytotoxicity of ALO on H1299 cells was measured using the LDH releasing assay (n = 3). B) Modulatory profiling of known small‐molecule cell death inhibitors in H1299 cells treated with ALO (200 µм, 24 h) (n = 3). C) Representative results of annexin V/7‐AAD staining in H1299 cells treated with ALO (0–200 µм) for 48 h (n = 3). D) The intracellular ROS level was measured in H1299 cells treated with ALO (200 µм) for 24 h (n = 3). E) Annexin V/7‐AAD staining was performed to estimate the ratio of cellular apoptosis in ALO‐treated H1299 cells transfected with *SQSTM1* siRNA (si*SQSTM1*) or negative control siRNA (siNC) (n = 3). F) Representative images and statistical analysis of TUNEL staining (green) in tumor sections from H1299‐derived subcutaneous tumor models and LLC‐derived subcutaneous tumor models in the ALO group and control (CT) group (n = 5). Scale bar: 50 µm. G) Immunoblotting assays were performed to assess LC3B‐II, SQSTM1, Bax and PARP levels in tumor tissues collected from H1299‐derived subcutaneous tumor models (n = 4) and LLC‐derived subcutaneous tumor models in the ALO group and CT group (n = 3). Data in A–E) are presented as mean ± SD, and *p* values were calculated using one‐way ANOVA. Data in F,G) are presented as mean ± SD, and *p* values were determined by two‐tailed unpaired Student's t‐test. ns, not significant; *p < 0.05, **p < 0.01, ***p < 0.001.

As ALO is identified as a late‐stage autophagy inhibitor, the next step is to elucidate whether ALO induces cytotoxicity in NSCLC cells by inferring with autophagic flux. First, ALO‐mediated cytotoxicity in NSCLC cell lines was analyzed in vitro by co‐culturing the H1299 and LLC cell lines with varying concentrations of ALO for diverse time periods. LDH release, an important indicator of cytotoxicity, was subsequently analyzed. The data revealed that ALO administration induced LDH release in H1299 and LLC cells in a time‐ and dose‐dependent manner (**Figure** **5**A; Figure S8A, Supporting Information), confirming that ALO induces cytotoxicity against NSCLC cells. Consistently, to ascertain whether autophagic death is responsible for the regulation of death process caused by ALO, the autophagy inhibitor SAR405 was applied in the co‐culture experiments. However, SAR405 could not block the cell death induced by ALO treatment (Figure 5B; Figure S8B, Supporting Information). Notably, the results revealed that apoptosis inhibitor Z‐VAD‐FMK, but not ferroptosis inhibitor (ferrostatin‐1) or necroptosis inhibitor (RIPK1 targeting necrostatin‐1),^[^ ^21^ ^]^ could block the cell death induced by ALO treatment (Figure 5B; Figure S8B, Supporting Information), indicating that apoptosis is responsible for the regulation of death process caused by ALO instead of autophagic death, ferroptosis or necroptosis. Additionally, Annexin V/7‐amino actinomycin D (7‐AAD) dual flow cytometry staining assay demonstrated a significant increase in both early and late apoptotic cells in NSCLC cell H1299, LLC and A549 cells following ALO treatment (Figure 5C; Figure S8C,D, Supporting Information), which was consistent with the increased apoptotic sub‐G1 population observed after ALO treatment (Figure 1F) and further confirmed that ALO induces cell apoptosis in NSCLC cells.

ALO inhibited autophagic flux, resulting in an increased level of SQSTM1 (Figure 3F; Figure S4E, Supporting Information). Considering that the accumulation of SQSTM1 has been reported to cause increased ROS production,^[^ ^22^ ^]^ and excessive ROS can damage cellular components, thereby promoting cell death processes such as apoptosis,^[^ ^23^ ^]^ we speculated that ALO‐induced cell apoptosis was likely triggered by SQSTM1 accumulation‐mediated excessive ROS production. To test this hypothesis, the ROS levels of H1299 and LLC cells were quantified upon ALO treatment. The results indicated that ALO significantly increased the production of ROS in H1299, LLC and A549 cells (Figure 5D; Figure S8E,F, Supporting Information). When Trolox, an inhibitor of ROS, was applied, ALO‐induced apoptosis was significantly reversed in both H1299 and LLC cells (Figure S8G, Supporting Information). Knocking down *SQSTM1* in cells by siRNA, resulted in significantly decreased ROS levels and apoptosis induced by ALO (Figure 5E; Figure S8H, Supporting Information). Furthermore, the combination of ALO with ATG7 silencing did not further increase ROS levels or apoptosis compared with ATG7 silencing alone in H1299 and LLC cells (Figure S9A–D, Supporting Information), indicating that ALO‐induced ROS accumulation and apoptosis in NSCLC cells are mediated by autophagy inhibition. Accordingly, ALO‐induced cell apoptosis is mainly induced by SQSTM1 accumulation‐mediated excessive ROS production.

Consistently, apoptosis induced by ALO was further detected in a subcutaneous tumor model in vivo (Figure 2A; Figure S2A, Supporting Information). The numbers of apoptotic cells, visualized by terminal deoxynucleotidyl transferase dUTP nick end labeling (TUNEL)‐positive cells, in H1299‐derived subcutaneous tumors and LLC‐derived subcutaneous tumors were significantly increased in the ALO‐treated group compared with the control (CT) group (Figure 5F). Immunoblotting analysis showed that the accumulation of LC3B‐II, SQSTM1, BCL2‐associated protein (Bax) and cleaved poly‐ADP ribose polymerase (PARP) in the tumor tissues were increased following ALO treatment compared with the CT group (Figure 5G), which suggested that ALO inhibited autophagic flux and induced apoptosis of NSCLC cells in vivo.

Interestingly, although ALO induced significantly lower levels of LC3B‐II and SQSTM1 accumulation in HBE cells compared with H1299 cells (Figure S10A, Supporting Information), analysis from RFP‐GFP‐LC3B reporter indicated that ALO inhibited the autophagic flux in HBE cells to some extent (Figure S10B, Supporting Information). However, flow cytometry analysis showed that ALO did not induce a significant increase in ROS levels and apoptosis in HBE cells (Figure S10C,D, Supporting Information). In contrast to tumor cells, normal cells have stronger antioxidant capacity, enabling them to better cope with oxidative damage.^[^ ^24^ ^]^ Consistently, the reduced glutathione (GSH) content was detected to evaluate the antioxidant capacity of cells. It is noteworthy that there was no significant change in the content of GSH in HBE cells with ALO treatment, while a remarkable decrease was observed in H1299 and LLC cells (Figure S10E, Supporting Information), indicating that HBE cells have a stronger antioxidant capacity than H1299 and LLC cells.

### 2.6 VPS4A Is Identified as the Direct Target of ALO

**Figure 6:**
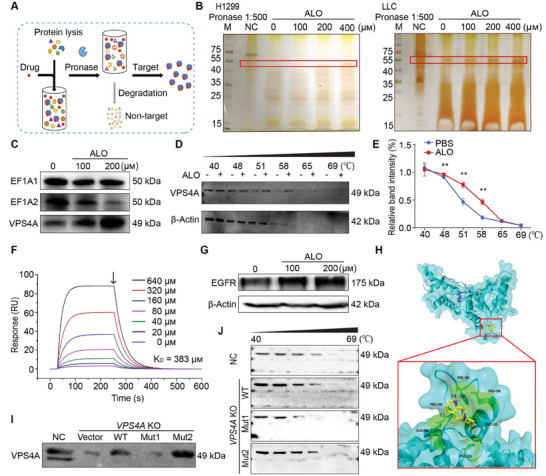
Identifying VPS4A as the direct target of ALO. A) Schematic diagram of drug affinity responsive target stability (DARTS) technology. B) The cellular target of ALO was identified using DARTS technology coupled with LC–MS/MS in H1299 and LLC cells. M, marker. C) VPS4A protein stability was increased upon ALO treatment in H1299 cell lysates. D,E) Cellular thermal shift assay (CETSA) assays confirmed the binding of ALO to VPS4A in H1299 cells, with β‐Actin serving as the internal control (n = 3). F) The binding of ALO to VPS4A was depicted through a surface plasmon resonance (SPR) sensorgram. The start of dissociation is indicated by the black arrow. G) Immunoblotting assays were performed to assess EGFR level in H1299 cells treated with ALO (200 µм, 48 h). H) Docking analysis of ALO covalent binding mode to yeast VPS4. I,J) *VPS4A* knockout (KO) H1299 cells were transfected with wild‐type (WT) and mutant VPS4A expressing plasmids, namely pENTER‐VPS4A^F153A; D263A^ (Mut1) and pENTER‐VPS4A^N262Y; G264F^ (Mut2). After 48 h, the cells were harvested, lysed, followed by treatment with ALO (200 µм) to assess the stability of VPS4A against pronase. H1299 cells served as the negative control (NC). The binding sites of VPS4A to ALO in H1299 cells were confirmed through DARTS assays I) and CETSA assays J). Data in E) are presented as mean ± SD, and *p* values were determined by two‐tailed unpaired Student's t‐test. *p < 0.05, **p < 0.01, ***p < 0.001.

To identify which molecules in NSCLC cells was targeted by ALO when exerting its antitumor efficacy, drug affinity responsive target stability (DARTS) technology was applied to identify the direct targets of ALO, as illustrated in **Figure** **6**A. The results indicated that a specific protein band with a size of ≈50 kDa increased in intensity upon ALO treatment of the cell lysate (Figure 6B). Peptides corresponding to proteins in this band were then identified by mass spectrometry, and ef1a1, ef1a2, VPS4A were selected for confirmation in immunoblotting analysis. The results demonstrated that VPS4A was a potential target of ALO, as it showed increased immunoblot signals after ALO treatment (Figure 6C). Next, cellular thermal shift assay (CETSA) was further performed to confirm the binding of ALO to VPS4A (Figure 6D,E).

Lung adenocarcinoma (LUAD) is the most common type of NSCLC. It has been reported that the low or absence of VPS4B makes cells more dependent on VPS4A.^[^ ^8^ ^]^ Intriguingly, the Cancer Genome Atlas (TCGA) and Clinical Proteomic Tumor Analysis Consortium (CPTAC) database showed that the mRNA and protein level of VPS4B in tumor tissues of patients with LUAD was significantly lower than normal tissues (Figure S11A,B, Supporting Information). In contrast, the same collection of patient samples demonstrated equal VPS4A mRNA level in normal tissues and LUAD tumor tissues (Figure S11A, Supporting Information). Additionally, immunoblotting assays revealed that the level of VPS4B in NSCLC cells was significantly lower than that in HBE cells, while there were no significant differences in VPS4A levels among these cells (Figure S11C, Supporting Information). Furthermore, overexpression of VPS4B in H1299 cells significantly reduced ALO‐induced increased LC3B‐II and SQSTM1 levels, as well as ROS accumulation and apoptosis (Figure S12A–C, Supporting Information). Meanwhile, ALO induced higher levels of LC3B‐II and SQSTM1, ROS accumulation, and apoptosis in VPS4B knocked‐down HBE cell (Figure S12D–F, Supporting Information). These results indicate that the imbalance between VPS4A and VPS4B provides an explanation for why ALO exhibits greater cytotoxicity on NSCLC cells than on HBE cells. Hence, VPS4A is a specific drug target for NSCLC therapy.

To further demonstrate the specific interaction of VPS4A with ALO, purified VPS4A protein was utilized to determine ALO binding ability by surface plasmon resonance (SPR) assays. The data revealed that ALO indeed bound to VPS4A with a K_D_ of 383 µм (Figure 6F), indicating that ALO has a moderate affinity for VPS4A. As a subunit of the ATPases Associated with diverse cellular Activity (AAA) + adenosine triphosphate (ATP) enzyme VPS4, VPS4A is necessary for the proper function of ESCRTs. Meanwhile, the degradation of epidermal growth factor receptor (EGFR) requires the ESCRT complex.^[^ ^25^ ^]^ The results showed that the level of EGFR was significantly increased in ALO‐treated H1299 cells (Figure 6G), providing further evidence for VPS4A being the cellular target of ALO.

Furthermore, to identify the specific binding sites of ALO within VPS4A, the two amino acid residues F153 and D263 were examined, which were predicted from the docking simulation for the binding of VPS4 in yeast (Figure 6H; Figure S13, Supporting Information). Subsequently, we generated wild‐type (WT) and mutant VPS4A expressing plasmids, namely pENTER‐VPS4A^F153A; D263A^ (Mut1) and pENTER‐VPS4A^N262Y; G264F^ (Mut2), and transfected them into *VPS4A* knockout (KO) H1299 cells to assess whether the WT and mutant VPS4A expressing plasmids could reintroduce the effects of ALO on biophysical interaction with VPS4A. DARTS assays with immunoblotting revealed that, compared with the WT pENTER‐VPS4A, the stability of VPS4A upon transfection of pENTER‐VPS4A^F153A; D263A^ against pronase decreased after treating with ALO (Figure 6I), confirming that F153 and D263 of VPS4A are indeed the binding sites for ALO. Additionally, CETSA was performed, further confirming the binding sites of VPS4A with ALO (Figure 6J).

### 2.7 ALO Induces SQSTM1 Accumulation by Targeting VPS4A

**Figure 7:**
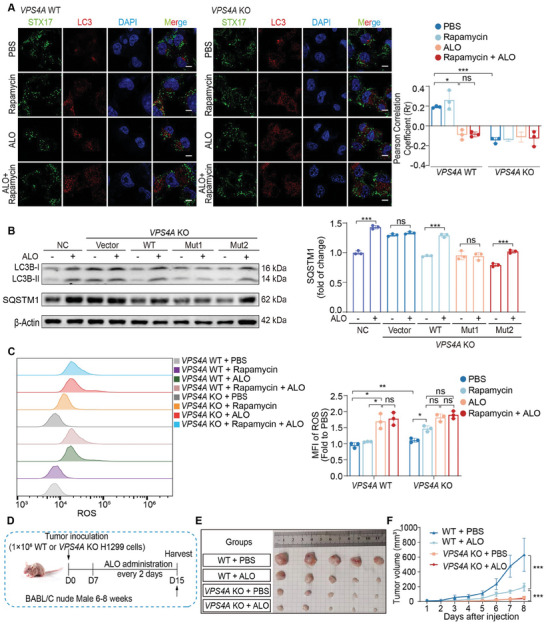
ALO exerts antitumor efficacy by targeting VPS4A. A) Fluorescence images of the colocalization of GFP‐STX17 and LC3 (red) in wild‐type (WT) H1299 and *VPS4A* knockout (KO) H1299 cells cultured in complete medium in the absence or presence of ALO (200 µм) for 2 h (n = 3). Scale bar: 10 µm. B) Immunoblotting assays were performed to assess LC3B‐II and SQSTM1 levels in H1299 and *VPS4A* KO H1299 cells treated with ALO (200 µм) for 2 h. Prior to ALO treatment, *VPS4A* KO H1299 cells were transfected with WT and mutant VPS4A expressing plasmids, pENTER‐VPS4A^F153A; D263A^ (Mut1) and pENTER‐VPS4A^N262Y; G264F^ (Mut2) and quantified by gray scale analysis (n = 3). H1299 cells were used as negative control (NC). C) The intracellular reactive oxygen species (ROS) level was determined in H1299 and *VPS4A* KO H1299 cells treated with ALO (200 µм) for 24 h in the absence or presence of 250 nм rapamycin (n = 3). D) Schematic diagram of tumor inoculation and injection protocol in H1299‐derived subcutaneous tumor mouse models. E) Images of tumors in WT and *VPS4A* KO H1299‐derived subcutaneous tumor mouse models (n = 5). F) Tumor growth curves of WT and *VPS4A* KO H1299‐derived subcutaneous tumor mouse models (n = 5). Data in A–F) are presented as mean ± SD, and *p* values were calculated using one‐way ANOVA. ns, not significant; *p < 0.05, **p < 0.01, ***p < 0.001.

Disfunction of VPS4A has been reported to be associated with unsealed autophagosomes, which may inhibit the fusion of autophagosomes with lysosomes through blocking the recruitment of STX17 to the autophagosomes membrane.^[^ ^5^ ^]^ Subsequently, whether ALO induced unsealed autophagosomes by targeting VPS4A in H1299 cells was investigated. Both ALO treatment and knockout of *VPS4A* led to a marked separation of LC3 puncta and STX17, which was distinctly different from changes induced by rapamycin (**Figure** **7**A). These findings indicate that ALO induces unsealed autophagosomes by targeting VPS4A, thereby interfering with STX17 recruitment onto the autophagosome membrane and inhibiting the fusion of autophagosomes with lysosomes. Additionally, no significant differences were observed in the colocalization of LC3 and LAMP1 in *VPS4A* KO H1299 cells in the presence or absence of ALO, confirming that ALO suppresses the fusion of autophagosomes with lysosomes by targeting VPS4A (Figure S14A, Supporting Information).

Subsequently, plasmids with or without VPS4A were transfected into *VPS4A* KO H1299 cells to determine whether the WT or mutant VPS4A expressing plasmids were able to reintroduce the ALO effects on H1299 cells. As compared with the WT pENTER‐VPS4A and pENTER‐VPS4A^N262Y; G264F^(Mut2), transfection with pENTER‐VPS4A^F153A; D263A^(Mut1) inhibited the increase of both LC3B‐II and SQSTM1 induced by ALO, further illustrating that ALO modulates the autophagy process by binding to the F153 and D263 sites of VPS4A (Figure 7B). To investigate whether ALO induces apoptosis in NSCLC cells by targeting VPS4A, the levels of ROS and apoptosis were assessed upon ALO treatment in both WT and *VPS4A* KO H1299 cells. It is noteworthy that knocking down VPS4A elevated ROS levels but was insufficient to induce apoptosis in H1299 cells (Figure 7C; Figure S14B, Supporting Information). Remarkably, rapamycin induced a comparable level of apoptosis in *VPS4A* KO H1299 cells compared with ALO‐treated H1299 cells, suggesting that ALO may not only target VPS4A, but also promote autophagy, ultimately leading to cell apoptosis. Taken together, ALO induces the formation of unsealed autophagosomes by targeting VPS4A, thereby inhibiting the fusion of autophagosomes and lysosomes, leading to the accumulation of SQSTM1 and increased ROS levels, ultimately triggering the occurrence of apoptosis.

To further explore whether ALO exhibits therapeutic effects on NSCLC tumor growth in vivo through targeting VPS4A, WT and *VPS4A* KO H1299 cells were subcutaneously injected into nude mice to establish a subcutaneous tumor model (Figure 7D). The data revealed no significant differences in body weight among the groups after ALO administration (data not shown). Importantly, the *VPS4A* KO H1299 cells‐induced subcutaneous tumors were notably suppressed, as evidenced by decreased tumor volume in mice compared with those in the WT group (Figure 7E,F). Moreover, ALO administration effectively inhibited WT H1299 xenografts, but showed no effect on *VPS4A* KO H1299 subcutaneous tumors (Figure 7E,F). These findings demonstrate that ALO exhibits therapeutic effects on NSCLC tumor growth in vivo specifically by targeting VPS4A.

### 2.8 ALO Enhances NK cell Cytotoxicity and Bispecific Antibody Efficacy in Tumor Treatment

**Figure 8:**
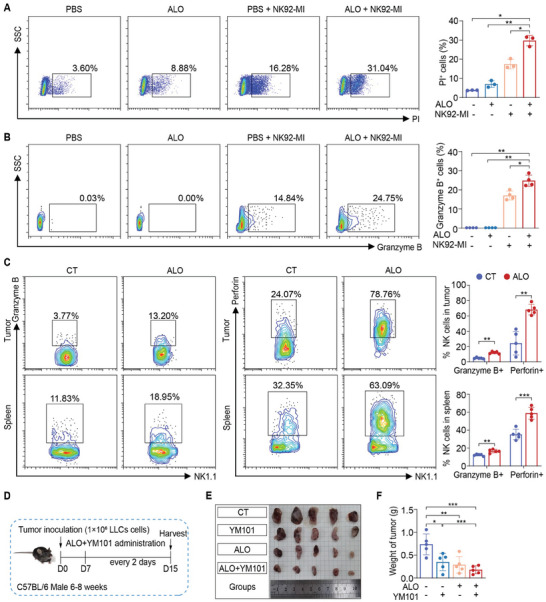
Evaluate the therapeutic efficacy of combination of ALO and YM101. A) H1299 cells were exposed to ALO (200 µм) in combination with NK‐92MI cells (Effector ‐to‐ target cell ratio = 5:1). Cell death was determined by propidium iodide (PI) staining. Cells were gated on FSC/SSC‐viability‐CD56‐negative (n = 3). B) Intracellular Granzyme B in H1299 cells was determined by flow cytometry. Cells were gated on FSC/SSC‐viability‐CD56‐negative (n = 4). C) Analysis of Granzyme B+ NK cells and Perforin+ NK cells in tumors and spleens of LLC‐derived subcutaneous tumor mouse models from each group was conducted by flow cytometry (n = 5). D) Schematic diagram of tumor inoculation and injection protocol in LLC‐derived subcutaneous tumor mouse models (n = 5). E) Image of LLC‐derived subcutaneous tumors. F) Weights of tumors in LLC‐derived subcutaneous tumor mouse models (n = 5). Data in A,B) and F) are presented as mean ± SD, and *p* values were calculated using one‐way ANOVA. Data in C) are presented as mean ± SD, and *p* values were determined by two‐tailed unpaired Student's *t*‐test. ns, not significant; *p < 0.05, **p < 0.01, ***p < 0.001.

ESCRT inhibition has been reported to increase cancer cell susceptibility to cytotoxic lymphocytes and elevate intracellular Granzyme B levels in cancer cells.^[^ ^26^ ^]^ The effects of ALO on the cytotoxicity of NK‐92MI cells NK‐92MI cells, a human‐derived natural killer cell line, were assessed. NK‐92MI cells were co‐cultured with H1299 cells for 24 h in the presence of ALO at an effector to target ratio of 5:1. PI uptake and intracellular Granzyme B were assessed using flow cytometry. The percentage of PI^+^ H1299 cells (**Figure** **8**A) and Granzyme B^+^ H1299 cells (Figure 8B) were both higher than that of the control group, indicating that ALO enhanced cytotoxicity of NK‐92MI cell to target cells.

Cytotoxic granules containing Granzyme B and perforin are crucial for the cytotoxic activity of NK cells in killing cancer cells.^[^ ^27^ ^]^ To investigate the modulation of ALO on the cytotoxic activity of NK cells in vivo, flow cytometry was used to analyze the phenotypes of NK cells in LLC‐derived subcutaneous tumor mouse models from each group (Figure 2A). Following ALO administration, the percentages of Granzyme B^+^ NK cells and Perforin^+^ NK cells (Figure 8C) significantly increased in tumors and spleens compared with the CT group, indicating that ALO may increase the cytotoxic activity of NK cells to exert antitumor efficacy.

YM101 is a bispecific antibody that blocks TGF‐β and murine PD‐L1, exhibiting potent antitumor activity in non‐inflamed cancers, like CT26 and B16 tumor models.^[^ ^28^ ^]^ The antitumor activity of the combination of ALO (50 mg kg^−1^) and YM101 (10 mg kg^−1^) was investigated in subcutaneous tumor mouse models in vivo (Figure 8D). The results indicated that the efficacy of the combination was significantly greater than either of ALO or YM101 monotherapy in LLC‐derived subcutaneous tumor mouse models, as evidenced by lower tumor weights compared with those in the ALO or YM101 group (Figure 8E,F). These findings suggests that the combination of ALO and immunotherapy may represent a promising strategy for NSCLC treatment.

## 3 Discussion

**Figure 9:**
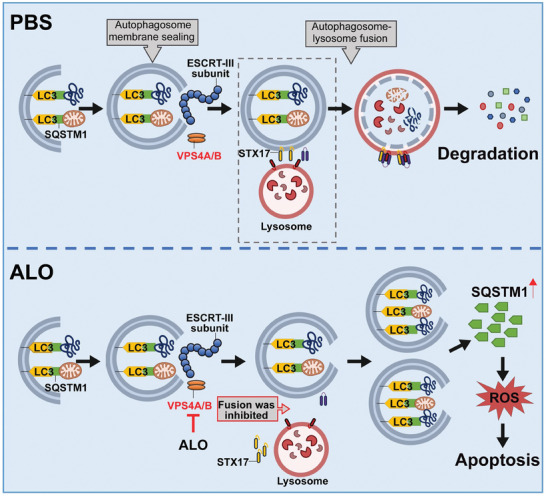
Schematic depiction about the antitumor effects of ALO in NSCLC.

Despite substantial advances in therapy for NSCLC in the past decade, the prognosis of patients with NSCLC remains unsatisfactory. Accumulating preclinical studies indicate that autophagy modulation is a promising strategy for lung cancer therapies.^[^ ^29^ ^]^ However, clinically approved autophagy inhibitors, such as hydroxychloroquine, have shown unsatisfactory efficacy in clinical trials. Previous study showed that ALO is a promising autophagy modulator,^[^ ^30^ ^]^ yet the underlying mechanism of its activity remains elusive. In this study, we comprehensively and systematically elucidated that ALO, by targeting VPS4A, induces unsealed autophagosomes and inhibits the fusion of autophagosomes with lysosomes, thereby inhibiting the autophagic flux accompanied with an accumulation of SQSTM1. The increased SQSTM1 levels induces ROS production, which subsequently triggers apoptosis of NSCLC cells (**Figure** **9**).

ALO was found to inhibit the proliferation and migration of NSCLC cells in vitro. The therapeutic efficacy of ALO was confirmed in different mouse models in vivo, including H1299 and LLC‐derived subcutaneous tumor models, lung metastasis tumor models and spontaneous lung adenocarcinoma models. These data suggest the clinical importance of ALO in NSCLC therapy. Flow cytometry analysis revealed that ALO arrested the cell cycle in G0/G1 phase, which is consistent with previous reports.^[^ ^31^ ^]^ In addition, transcriptome analysis showed that ALO decreased the expression of cell cycle related gene, including CCND1 and CDK4, in NSCLC cells. Considering that cyclin D1‐CDK4 complexes phosphorylate retinoblastoma 1 and regulate the cell‐cycle during G1/S transition,^[^ ^32^ ^]^ this may provide an explanation for the G0/G1 phase arrest induced by ALO.

Plenty of evidence indicates that autophagy has great potential in promoting tumor proliferation, progression and viability, suggesting a promising prospect for autophagy inhibition in cancer therapies.^[^ ^1^ ^]^ Intriguingly, the autophagy pathway was enriched in KEGG pathway enrichment analysis after ALO treatment. Likewise, a large accumulation of autophagic vesicles were observed in ALO‐treated cells under transmission electron microscopy. SQSTM1 is commonly used to indirectly reflect autophagic flux, a critical cellular process to monitor the dynamic process of autophagy.^[^ ^18^ ^]^ Immunoblotting assays demonstrated that SQSTM1 levels were upregulated in NSCLC cells after the administration of ALO, suggesting that autophagic flux was blocked upon ALO treatment. Additionally, co‐incubation of cells with ALO evoked a more pronounced accumulation of LC3B‐II than autophagy inducer rapamycin which suggested ALO functions as an autophagy inhibitor.

Most of the autophagy inhibitors discovered so far either impair lysosomal hydrolysis or affect lysosomal pH.^[^ ^33^ ^]^ However, data from mCTSD detection and acidophilic dye staining suggested that ALO did not affect lysosomal pH or lysosomal proteolytic activity in both H1299 and LLC cells. Consistently, the co‐localization of autophagosome and lysosomes was analyzed, and GFP‐LC3B puncta were not co‐localized with LysoTracker Red after ALO treatment, suggesting that ALO treatment might impair the fusion of autophagosome with lysosomes. Taken together, these data clearly support that ALO serves as a late‐stage autophagy inhibitor, suppressing the fusion of autophagosomes with lysosomes. This inhibitory effect is independent of an impairment of lysosomal functions, and ultimately inhibits the autophagic flux.

Both in vivo and in vitro analyses indicate that ALO inhibits autophagy and promotes apoptosis in NSCLC cells. Hence, it is worth exploring whether the apoptosis induced by ALO is related to autophagy modulation. ROS are widely generated in various redox reactions, and increased ROS production has been shown to promote anti‐tumorigenic signaling by initiating oxidative stress‐induced tumor cell death.^[^ ^34^ ^]^ The in vitro data showed that ALO induced the upregulation of ROS production in NSCLC cells. Consistently, scavenging ROS significantly reversed increased apoptotic cells in ALO‐treated NSCLC cells, indicating that ROS is the key determinant of ALO‐induced apoptosis. Nevertheless, the exact mechanism of how ALO induces ROS release remains a question. Mounting evidence shows that ROS levels are closely correlated with the intracellular autophagy, and actually both processes work tightly together to maintain cellular homeostasis.^[^ ^35^ ^]^

The SQSTM1 is a selective autophagy receptor that is recruited to phagophores and constitutively degraded during autophagy.^[^ ^18^ ^]^ Notably, SQSTM1 levels were obviously upregulated upon ALO treatment in both H1299 and LLC cells, which positively correlated with ROS level observed upon ALO treatment. This pattern is consistent with previous reports indicating that the accumulation of SQSTM1 cause an increase in ROS production.^[^ ^22^ ^]^ Knocking down *SQSTM1* by siRNA significantly inhibited elevated ROS levels and reversed cell apoptosis induced by ALO, suggesting that elevated ROS production in ALO‐treated cells is induced by the accumulation of SQSTM1 due to ALO‐mediated inhibition of autophagic flux.

VPS4A, a member of the AAA adenosine triphosphatase family, is a key regulator of exosome biogenesis through modulating the ESCRTs machinery.^[^ ^5^ ^]^ In this study, VPS4A was identified as the main ALO target as evidenced by DART, mass spectrometry, CETSA, SPR assays, and docking analysis. ESCRTs play a vital role in membrane remodeling, and deletion of the ESCRT complex in mammalian cells has been shown to inhibit the degradation of proteins such as EGFR.^[^ ^5^, ^36^ ^]^ As expected, a substantial accumulation of EGFR was observed in ALO‐treated cells, suggesting that ALO might interrupt VPS4A‐mediated downstream effects of the ESCRT. As an indispensable subunit of ESCRTs, VPS4A has been reported to be closely associated with phagophore closure. Specifically, that unsealed autophagosomes prevents the recruitment of STX17 onto the autophagosomes membrane, a process crucial for autophagosome‐lysosome fusion.^[^ ^5^, ^36^ ^]^ Consistently, both ALO treatment and *VPS4A* knockout induced the formation of unsealed autophagosomes, decreased the recruitment of STX17 to autophagosomes membrane, inhibited the fusion of autophagosomes and lysosomes, and led to the accumulation of SQSTM1 and ROS levels in H1299 cells. While *VPS4A* knockout alone was insufficient to induce apoptosis in H1299 cells, rapamycin induced a comparable level of apoptosis in *VPS4A* KO H1299 cells compared with ALO‐treated H1299 cells. The findings indicate that ALO may function not only as an autophagy inhibitor by targeting VPS4A but also as an autophagy activator. In addition, ALO administration effectively inhibited the growth of H1299‐derived subcutaneous tumor**s** without affecting *VPS4A* KO H1299‐derived subcutaneous tumor**s**, confirming its therapeutic efficacy in inhibiting NSCLC tumor growth in vivo by targeting VPS4A.

When cytotoxic lymphocytes attacks cancer cells, cytolytic protein induces the loss of plasma membrane integrity, leading to an ESCRT‐dependent membrane repair response.^[^ ^26b^ ^]^ Consistently, ESCRT inhibition induced enhances the susceptibility of cancer cells to cytotoxic lymphocytes. Intriguingly, H1299 cells indeed became more sensitive to NK cells after treatment of ALO in vitro. To further evaluate the therapeutic potential of combining ALO with immunotherapy, the efficacy of the ALO and YM101 combination was examined in vivo. The data showed that the combination was significantly more effective than either ALO or YM101 monotherapy in LLC‐derived subcutaneous tumor models, indicating that the promising anti‐tumor efficacy of combining ALO with immunotherapy deserves further exploration for NSCLC treatment. Additionally, the results indicated that ALO might enhance the cytotoxic activity of NK cells to exert antitumor efficacy in vivo. Nevertheless, further in vivo and in vitro experiments are required to verify whether ALO can directly regulate immune cells to exert its anti‐tumor effects, which is also the focus of our next research direction.

Transcriptomic data indicate that ALO treatment increases mRNA levels of LC3 and SQSTM1 in NSCLC cells. Both oxidative stress and the SQSTM1 accumulation have been shown to elevate SQSTM1 transcription levels,^[^ ^37^ ^]^ indicating that the increase in SQSTM1 transcription levels induced by ALO may result from elevated intracellular ROS levels and SQSTM1 accumulation. Notably, co‐incubation of cells with ALO evoked a more substantial accumulation of LC3B‐II than late‐stage autophagy inhibitor bafA1 alone, suggesting that ALO may induce autophagosome formation. In addition, there was no increase in LC3B‐II levels with ALO and rapamycin co‐treatment compared with ALO alone in NSCLC cells, indicating that ALO treatment might also induce autophagy. Previous work by Lin et al. demonstrated that ALO induces the production of autophagosome‐like vacuoles in cells.^[^ ^38^ ^]^ Notably, both in vitro and in vivo analyses revealed that ALO treatment interferes with the activation of the Akt/mTOR pathway in NSCLC cells, while levels of Beclin‐1 and ATG7 increase, suggesting that ALO treatment induces autophagosome generation. However, the regulation of the PI3K/Akt/mTOR signaling pathway by ALO seems to be context‐dependent. In various disease models, some studies have demonstrated that ALO activates the PI3K/Akt pathway,^[^ ^39^ ^]^ while others indicate that ALO suppresses it.^[^ ^40^ ^]^ Consequently, further investigation is necessary to elucidate whether ALO directly regulates the PI3K/Akt/mTOR signaling pathway in NSCLC cells and to understand the mechanisms involved.

As an important issue, the side effects of ALO were assessed systematically in the present study. Interestingly, while ALO inhibits autophagic flux in HBE cells, its cytotoxicity to HBE cells is much lower than that to NSCLC cells. Consequently, the discrepancy of the mechanism between NSCLC and HBE cells was investigated. First, the content of VPS4B in NSCLC cells is significantly lower than that in HBE cells, rendering NSCLC cells more reliant on VPS4A. As a result, when ALO targets VPS4A inhibition, the impact on the autophagic flux in NSCLC cells surpasses that in HBE cells; second, HBE cells exhibit stronger antioxidant capacity compared with NSCLC cells, thus reducing oxidative damage and apoptosis caused by ALO. Additionally, results from histopathological analysis, biochemical analysis, and analysis of the number and proportion of immune cells demonstrated the therapeutic potential of ALO in inhibiting NSCLC growth without obvious side effects in vivo. Consistently, the safety of ALO for in vivo application has also been reported in accumulating studies.^[^ ^41^ ^]^ Nevertheless, minimizing the side effects of autophagy regulation on normal cells remains a pivotal concern. First, a broad understanding of the cellular events such as autophagy levels, ROS levels, and VPS4A and VPS4B levels can enable the strict screening of NSCLC patient populations suitable for ALO treatment. Moreover, considering the advantages of nanoparticles in enhancing bioavailability, reducing toxic side effects and organ targeting,^[^ ^42^ ^]^ ALO‐loaded nanoparticles are under development for localized therapy of NSCLC by nebulization administration, which is expected to further enhance the safety and specificity of ALO.

## 4 Conclusion

In summary, ALO is identified as a novel late‐stage autophagy inhibitor as verified by both in vitro and in vivo analyses. Mechanistically, ALO interacts with the F153 and D263 amino acids of VPS4A, leading to the formation of unsealed autophagosomes, which inhibits the fusion of autophagosomes and lysosomes. Furthermore, ALO‐mediated blockage of autophagic flux induces the accumulation of SQSTM1, which promotes excessive ROS production, resulting in apoptosis of NSCLC cells. Additionally, ALO enhances the efficacy of bispecific antibody in treating LLC‐derived subcutaneous tumors, suggesting its potential as a promising adjuvant in tumor immunotherapy. Taken together, this study identified ALO as a novel late‐stage autophagy inhibitor that triggers apoptosis of NSCLC cells by targeting VPS4A.

## 5 Experimental Section

### Materials

ALO was purchased from Shanghai yuanye Bio‐Technology and dissolved in Phosphate Buffer Saline (PBS) to make a stock solution at 20 mM, which was stored at −80 °C and diluted with PBS to certain concentrations before used. Puromycin (A1113803) were purchased from Thermo Fisher. Kanamycin (HY‐16566), Ferrostatin‐1 (HY‐100579), SAR405 (HY‐12481), Z‐VAD‐FMK (HY‐16658B), necrostatin‐1 (HY‐15760), rapamycin (HY‐10219), Trolox (HY‐101445) were purchased from Med Chem Express. Tat‐beclin 1 (S8595) was purchased from Selleck Chemicals. Propidium Iodide (PI, ST511), Goat Serum (C0265), Reactive Oxygen Species Assay Kit (S0033M), One Step TUNEL Apoptosis Assay Kit (C1088) and LysoTracker Red (C1046) were purchased from Beyotime Biotechnology. LysoSensor Green DND‐189 (40767ES50) and Hoechst 33 342 (40731ES10) were purchased from Yeasen Biotechnology. Ampicillin (A5354) was purchased from Sigma–Aldrich. h*VPS4A* gRNA (GTTGGGCTTCTCCATCACGA), pENTER‐VPS4A‐mut1, pENTER‐VPS4A‐mut2, pcDNA3.1‐3*Flag‐hSTX17, pcDNA3.1‐3*FLAG‐VPS4B and specific siRNA against SQSTM1, ATG7 and VPS4B were constructed by PAIVIBIO.

### Cell Culture

All cell lines were purchased from the American Tissue Culture Collection, including human NSCLC cell line H1299 (CRL‐5803), murine NSCLC cell line LLC (CRL‐1642), HBE (CRL‐2741), A549 (CCL‐185) and NK‐92MI cells (CRL‐2408). All cells were cultured in a 37 °C incubator with 5% CO_2_, using complete medium supplemented with 10% fetal bovine serum (FBS, Gibco, 10 099), and 100 U mL^−1^ Penicillin‐Streptomycin (Gibco, 15 140 148). H1299 and HBE cells were cultured in RPMI‐1640 medium (Gibco, 11 875 119), LLC cells were cultured in Dulbecco's modified Eagle medium (DMEM, [Gibco, 11 965 092]), and NK‐92MI cells were cultured in NK‐92MI specific complete medium (Procell Life Science & Technology, CM‐0533).

### Measurement of the Cell Inhibition Rate and Lactate Dehydrogenase Assay

H1299 cells or LLC were seeded onto 96‐well culture dishes. Upon reaching 80% confluency, cells were treated with PBS (Gibco, 10 010 023) or various concentrations of ALO for 24, 48, and 72 h. Subsequently, the cell culture supernatant was collected and centrifugated at 400 g for 10 min. The suspension was transferred into a new 96‐well plate for LDH releasing assay, following the manufacturer's protocols (Beyotime Biotechnology, C0017). The absorbance of the reaction mixture was measured at 340 nm using an EpochTM spectrophotometer (America). Cell viability was assessed using a Cell Counting Kit‐8 (Beyotime Biotechnology, C0038).

### Colony Formation Assay

The colony formation assay was performed as described previously.^[^ ^43^ ^]^ In brief, lung cancer cells were seeded in 10 cm dishes at a density of 1000 cells per well and treated with varying concentrations of ALO for 10 days. After washing twice with PBS, the cells were fixed with methanol for 15 min at room temperature, followed by staining with 1% crystal violet (Beyotime Biotechnology, C0121) for 10 min. Colony numbers were then counted for analysis.

### Flow Cytometric Cell Cycle and Apoptosis Analysis

The cells were fixed with 70% alcohol for 1 h at −20 °C and then stained with a cell cycle staining kit (Multiscience, CCS012) at room temperature. Cell cycle distribution was determined using a BD AccuriTM C6 Analyzer (America). For the cell apoptosis assay, cells were collected and analyzed by an Annexin V‐FITC/7‐AAD Apoptosis Detection Kit (BD Biosciences, 559 763) or Annexin V/PI Apoptosis Detection kit (Abbkine Scientific Co., KTA0002).

### Transwell and Wound‐Healing Assays

A cell invasion assay was performed as described previously.^[^ ^44^ ^]^ Briefly, an 8 µm pore‐size invasion chamber coated with Matrigel (Beyotime Biotechnology, C0371) was utilized. Cells suspended in serum‐free medium at a concentration of 1 × 10^5^ mL^−1^ were seeded into the upper chamber, while the lower compartment was filled with complete medium. After 24 h, the invading cells adhering to the bottom surface of the chamber membrane were fixed with methanol and then stained with 0.2% crystal violet. Images of three different fields were captured from each membrane, and the number of invading cells was counted. Uncoated chambers were used in the migration assay with a similar method. For the wound‐healing assay, a monolayer of cells at 95% confluence was scratched using a sterile plastic tip, followed by incubation in serum‐free medium.

### Reverse Transcription and Quantitative Real Time Polymerase Chain Reaction (qRT‐PCR)

The qRT‐PCR was performed as described previously.^[^ ^45^ ^]^ Total RNA was extracted using the TRIzol reagent (Life Technologies, 15 596 026). Subsequently, the cDNA synthesis was performed with a Primescript first‐strand cDNA synthesis kit (Vazyme Biotechnology, R323‐01). The qPCR assay was utilized to quantify the mRNA levels of *MMP3, MMP9, SNAI2*, *VIM*, *CDH1*, and *CDH2* and of *ACTB* (as an internal control) using the SYBR Green Supermix (Vazyme Biotechnology, Q111–02/03). The primer sequences are listed in **Table** **1**.

**Table 1:** Primer sequences used for quantitative real‐time PCR.

Species	Genes		Primer sequences (5′–3′)
Human			
	*ACTB*	Sense	CACCATTGGCAATGAGCGGTTC
		Anti‐sense	AGGTCTTTGCGGATGTCCACGT
	*CDH1*	Sense	GCCTCCTGAAAAGAGAGTGGAAG
		Anti‐sense	TGGCAGTGTCTCTCCAAATCCG
	*CDH2*	Sense	CCTCCAGAGTTTACTGCCATGAC
		Anti‐sense	GTAGGATCTCCGCCACTGATTC
	*MMP3*	Sense	CACTCACAGACCTGACTCGGTT
		Anti‐sense	AAGCAGGATCACAGTTGGCTGG
	*MMP9*	Sense	GCCACTACTGTGCCTTTGAGTC
		Anti‐sense	CCCTCAGAGAATCGCCAGTACT
	*SNAI2*	Sense	ATCTGCGGCAAGGCGTTTTCCA
		Anti‐sense	GAGCCCTCAGATTTGACCTGTC
	*VIM*	Sense	AGGCAAAGCAGGAGTCCACTGA
		Anti‐sense	ATCTGGCGTTCCAGGGACTCAT
Mouse	*Actb*	Sense	GTGACGTTGACATCCGTAAAGA
		Anti‐sense	GTAACAGTCCGCCTAGAAGCAC
	*Cdh1*	Sense	GGTCATCAGTGTGCTCACCTCT
		Anti‐sense	GCTGTTGTGCTCAAGCCTTCAC
	*Cdh2*	Sense	CCTCCAGAGTTTACTGCCATGAC
		Anti‐sense	CCACCACTGATTCTGTATGCCG
	*Mmp3*	Sense	CTCTGGAACCTGAGACATCACC
		Anti‐sense	AGGAGTCCTGAGAGATTTGCGC
	*Mmp9*	Sense	GCTGACTACGATAAGGACGGCA
		Anti‐sense	TAGTGGTGCAGGCAGAGTAGGA
	*Snai2*	Sense	TCTGTGGCAAGGCTTTCTCCAG
		Anti‐sense	TGCAGATGTGCCCTCAGGTTTG
	*Vim*	Sense	CGGAAAGTGGAATCCTTGCAGG
		Anti‐sense	AGCAGTGAGGTCAGGCTTGGAA

### Western Blotting Assay

Cell lysates were prepared in lysis buffer (Beyotime Biotechnology, P0013), and the BCA kit (Beyotime Biotechnology, P0011) was used to determine the protein concentration. The samples were loaded onto a sodium dodecyl sulfate (SDS)‐PAGE gel and subsequently electro‐transferred to a PVDF membrane (Thermo Fisher Scientific, 88 518). After blocking with 5% nonfat milk for 1 h, the membrane was incubated with antibodies to β‐Actin (ABclonal Technology, AC004; 1:500), lC3B (ABclonal Technology, A19665; 1:500), SQSTM1 (ABclonal Technology, A19700; 1:500), CTSD (Cell Signaling Technology, 74 089; 1:500), Ef1A1 (ABclonal Technology, A23515; 1:500), Ef1A2 (ABclonal Technology, A7327; 1:500), VpS4A (ABclonal Technology, A7096; 1:500), VPS4B (ABmart Technology, PH5915S; 1:500), EGFR (ABclonal Technology, A11351; 1:500), PaRP1 (ABclonal Technology, A0942; 1:500), Bax (ABclonal Technology, A0207; 1:500), AKT1 (ABclonal Technology, A5523; 1:500), p‐AKT1‐t308 (ABclonal Technology, AP0304; 1:500), mTOR (ABclonal Technology, A2445; 1:500), p‐mTOR‐S2448 (ABclonal Technology, AP0094; 1:500), Beclin‐1 (ABclonal Technology, A21191; 1:500), ATG7 (Abmart Technology, TN23498; 1:500), MMP9 (Proteintech Technology, 27306‐1‐AP, 1:500), SNAI2 (Proteintech Technology, 12129‐1‐AP, 1:500), Vimentin (Proteintech Technology, 10366‐1‐AP, 1:500), Cadherin‐2 (Proteintech Technology, 22018‐1‐AP, 1:500), and Cadherin‐1 (Proteintech Technology, 20874‐1‐AP, 1:500). Subsequently, the membrane was incubated with horseradish peroxidase (HRP)‐conjugated goat anti‐mouse (Beyotime Biotechnology, A0216) or anti‐rabbit secondary antibodies (Beyotime Biotechnology, A0208) for 1 h at room temperature. The reaction was visualized using Clarity Western ECL substrate (Bio‐Rad, 1 705 060) and detected by exposure to autoradiographic film.

### Transfection

Transfection was performed using Lipofectamine 3000 (Thermo Fisher Scientific, L3000015) according to the manufacturer's instructions. Briefly, cells at 60%–70% confluence were transfected with plasmids or siRNA using Lipofectamine 3000 in Opti‐MEM (Gibco, 31 985 070). After incubating with transfection complexes for 72 h, cells were harvested.

### Generation of Stable Cell Lines

To generate a stable cell line expressing the RFP‐GFP‐LC3B reporter or GFP‐LC3B reporter, lentiviruses carrying stubRFP‐senseGFP‐LC3B and GFP‐LC3B constructs were generated by Shanghai GeneChem and used for the generation of stable cell lines by lentivirus‐mediated transfection. H1299, LLC and HBE cells at a confluency of 20%–30% were transfected by adding 10 µL 1 × 10^8^ TU mL^−1^ lentivirus and 8 µL P reagent in a 48‐well dish with 1 × 10^5^ cells per well. After 72 h of transfection, puromycin was added into the culture medium, and resistant clones were subsequently screened.

### GSH Assay

GSH content were determined using a GSH assay kit (Nanjing Jiancheng, A006‐2‐1). In short, H1299, LLC and HBE cells were cultured in a 12‐well plate with a density of 1 × 10^5^ cells per well. After incubation with ALO for 24 h, the cells were harvested and resuspended in PBS, and then lysed by ultrasound. The supernatant of lysate was collected by centrifugation to detect the level of GSH in cells.

### SPR Assay

The COOH chip was installed according to the operation manual of the Open SPRTM instrument (Canada). The analyte was diluted with 1% DMSO and loaded at a speed of 20 µL min^−1^. The interaction time between protein and ligand was 240 s, and the natural dissociation time was 300 s. The analysis was conducted using the One‐to‐One analysis model with Trace Drawer software.

### Transmission Electron Microscopy

H1299 cells or LLC were seeded into 100‐mm culture dishes. Upon reaching 80% confluency, cells were exposed to PBS or ALO for 24 h. Then the cells were fixed, dehydrated, embedded, sectioned, and stained as previously described.^[^ ^46^ ^]^ Finally, the ultrathin sections of these samples were observed under a JEM‐1230 transmission electron microscopy (Japan).

### Double‐Immunofluorescence Staining

H1299 cells or LLC were plated on 48‐well culture dishes. Upon reaching 30% confluency, cells were treated with PBS, ALO, or Bafilomycin‐A1(Med Chem Express, HY‐100558) for 2 h. After treatment, cells were fixed in 4% paraformaldehyde for 15 min at 4 °C and blocked in 5% BSA for 30 min. Subsequently, cells were incubated with antibodies to LAMP1 (Sigma–Aldrich, L1418) and LC3 (MBL Beijing Biotech, M152‐3) overnight at 4 °C, followed by incubation with the Anti‐mouse IgG (H+L′, F(ab″)2 Fragment (Alexa Fluor 488 Conjugate, [Cell Signaling Technology, 4408]) or Anti‐rabbit IgG (H+L′, F(ab″)2 Fragment (Alexa Fluor 594 Conjugate, [Cell Signaling Technology, 8889]). The samples were then covered with Antifade mounting medium with DAPI (Beyotime Biotechnology, P0131). Fluorescence images were captures using Olympus FLUOVIEW FV3000 (Japan). Multiple fields of view (>5 regions) were analyzed on the confocal laser‐scanning microscope for each labeling condition, and representative results were shown.

### RNA Library Construction and Sequencing

The total RNA was extracted by TRIzol reagent. Then, paired‐end sequencing was conducted on an Illumina HiSeq 4000 at LC‐BIO Technologies (China), following the vendor's recommended protocol.

### DARTS

The cell lysates were prepared with lysis buffer, and the concentration was determined as described above.^[^ ^47^ ^]^ Subsequently, the cell lysates were mixed with ALO or PBS (up to 200 µм) for 1.5 h at room temperature for the binding reaction. Pronase (Sigma–Aldrich, 10 165 921 001) was then added, followed by incubation for 30 min. The samples were loaded on SDS/PAGE, and the gel was stained with PAGE Gel Silver Staining Kit (Solarbio Life Sciences, G7210) for mass spectrometry identification.

### Immunohistochemistry Staining

Tumor slices were socked in 10% goat serum for 1 h. Then, the slices were incubated with ki67 antibody (ABclonal Technology, A20018) overnight at 4 °C, followed by incubation with secondary antibody at room temperature for 1.5 h. Finally, the slices were stained using a DAB kit (Thermo Fisher, 34 002).

### Data Collection

TCGA LUAD data was retrieved using the Xena browser (https://xenabrowser.net).^[^ ^48^ ^]^ Protein levels in normal and primary tumor tissues were downloaded from the UALCAN portal (https://ualcan.path.uab.edu/cgi‐bin/ualcan‐res.pl) using the CPTAC database.^[^ ^49^ ^]^

### Flow Cytometry

Flow cytometry was performed as described previously.^[^ ^50^ ^]^ Red blood cells were lysed by RBC Lysis Buffer (Biolegend, 420 301), followed by washing with FACS buffer (PBS with 2% FBS). Cells were then resuspended and stained with indicated antibodies diluted by FACS buffer for 30 min at 4 °C for respective cell type analysis. For staining of IFN‐γ, IL‐17A, Granzyme B and perforin, cells were incubated and stimulated with 200 ng ml^−1^ Phorbol myristate acetate (Enzo Life Sciences, BML‐PE160‐0005), 1 µg ml^−1^ ionomycin (Enzo Life Sciences, ALX‐450‐007‐M001), and 1 µg ml^−1^ brefeldin A (eBioscience, 00‐4506‐51) at 37 °C for 6 h. Then, cultured cells were collected, washed, and stained for surface markers including Fixable Viability Dye (Biolegend, 423 101), FITC anti‐mouse CD45.2 antibody (eBioscience, 11‐0454‐85), FITC anti‐mouse CD45.2 antibody (BD Biosciences, 553 772), FITC anti‐mouse CD4 antibody (BD Biosciences, 553 046), APC anti‐mouse CD3 antibody (BD Biosciences, 553 066), FITC anti‐human CD56 antibody (BD Biosciences, 562 794) and PE‐Cy7 anti‐mouse NK1.1 antibody (BD Biosciences, 552 878). Cells were then fixed and permeabilized with fixation and permeabilization buffer (Thermo Fisher, 88‐8824‐00) at room temperature for 30 min, followed by staining with APC anti‐mouse IFN‐γ antibody (Biolegend, 505 809), PerCP‐Cy5.5 anti‐mouse IL‐17A antibody (BD Biosciences, 560 666), APC anti‐human/mouse Granzyme B antibody (Biolegend, 372 204) and PE anti‐mouse Perforin antibody (Biolegend, 154 306). For Foxp3 staining, cells were stained for surface markers, followed by fixation and permeabilization with fixation and permeabilization buffer. Subsequently, cells were stained with PE anti‐mouse Foxp3 antibody (BD Biosciences, 560 408). The antibodies used for cell type analysis also include PE‐Cy7 anti‐mouse CD8a antibody (Biolegend, 100 721), PE‐Cy7 anti‐mouse CD11c antibody (BD Biosciences, 558 079), PE‐Cy7 anti‐mouse CD4 antibody (Biolegend, 100 527), PerCP‐Cy5.5 anti‐mouse CD45.2 antibody (BD Biosciences, 552 950), APC anti‐mouse CD25 antibody (BD Biosciences, 557 192), APC anti‐mouse CD115 antibody (BD Biosciences, 567 027), APC‐Cy7 anti‐mouse CD62L antibody (Biolegend, 104 427), APC‐Cy7 anti‐mouse Ly‐6G antibody (Biolegend, 127 623), APC‐Cy7 anti‐mouse CD3 antibody (Biolegend, 100 221), PE anti‐mouse CD19 antibody (Biolegend, 115 507), PE anti‐mouse Ly‐6C antibody (BD Biosciences, 560 592), PE anti‐mouse F4/80 antibody (BD Biosciences, 565 410), BV650 anti‐mouse I‐A/I‐E antibody (BD Biosciences, 743 873), APC anti‐mouse CD206 antibody (Invitrogen, 17‐2061‐82), and eFlour450 anti‐mouse CD11b antibody (Invitrogen, 48‐0112‐82). Flow gating strategies for each cell population were shown in Figure S15 (Supporting Information).

### Animal Treatment

Five‐week‐old C57BL/6 and BALB/c nude mice were purchased from Beijing Vital River Laboratory Animal Technology Co., Lt, and *Kras^G12D^; Trp53^fl/fl^* mice were purchased from Shanghai Model Organisms Center, Inc. All mice were housed in specific pathogen‐free conditions at the Experimental Animal Center in the Institute of Medicine in Tongji medical school (Hubei Province, China). Mice were subcutaneously injected with H1299 or LLC cells (1 × 10^6^) at the right scapula. Seven days post‐cell implantation, palpable tumors were observed, and the mice were then randomly divided into groups. Six‐week‐old *Kras* ^G12D^;* Trp53^fl/fl^* mice were inoculated with 2 × 10^11^/50 µL Cre‐AAV (OBiO Technology Corp., Ltd.) by tracheal administration to obtain *Kras* ^G12D^; *Trp53* ^−/−^ mice and promote the spontaneous formation of lung adenocarcinoma. ALO or YM101 (Wuhan YZY Biopharma) were intraperitoneally injected every 2 days, with PBS serving as a control. Starting from the initial injection, the tumor size was measured every 2 days, and tumor volumes were calculated as tumor length × (square of width)/2. At the end of the experiment, all mice were sacrificed, and the tumors were resected immediately and photographed. The weight of each tumor was recorded. BMs, spleens, MLNs, and thymuses were collected from the mice for flow cytometry analysis. BMs were collected from the tibia and fibula of mice. All procedures were in accordance with the Guide for the Care and Use of Laboratory Animals published by the US National Institutes of Health (NIH Publication No. 85–23, revised 1996) and were approved by the Committee for Animal Research of Huazhong University of Science and Technology (Wuhan, China) (IACUC‐2697).

### Histopathology Scoring

Histopathology scoring was performed in a blinded manner as previously reported.^[^ ^51^ ^]^ Briefly, mice tissues were collected and fixed in 10% neutral buffered formalin (Solarbio Life Sciences, G2161). The fixed tissues were then embedded in paraffin, sectioned at 5 µm, and stained with hematoxylin and eosin (H&E) for light microscopy. Liver pathology was graded based on the average number of inflammatory foci in non‐overlapping fields (200×): 0 (none), 1 (1–3), 2 (4–7) and 3 (>7).^[^ ^52^ ^]^ The histopathology scoring of kidneys, evaluating the extent of loss of brush border, tubular necrosis and dilatation, was as follows: 0 (none), 1 (≤10%), 2 (11%–25%), 3(26%–45%), 4 (46%–75%), and 5 (≥76%).^[^ ^51^ ^]^ The extent of pulmonary hemorrhage was graded as 0 (none), 1 (single focus), 2 (multiple foci), and 3 (locally extensive).^[^ ^52^ ^]^

### Statistical Analysis

All experiments were performed in duplicate and repeated at least 3 times. Data were expressed as means ± standard deviation (SD). The exact sample size of each experimental group was shown in every figure legend. The differences between two groups were analyzed by two‐tailed unpaired Student's t‐test, and the differences among three or more groups were analyzed by one‐way variance (ANOVA) with post hoc tests. Statistical analysis was performed using SPSS (version.17.0). Differences at *p* < 0.05 were considered statistically significant: ns, not significant; *p < 0.05, **p < 0.01, ***p < 0.001.

## Conflict of Interest

The authors declare that there are no conflicts of interest.

## Author Contributions

W.G., H.Z., and J.W. contributed equally to this work. W.G. and H.Z. performed most of the experiments, analyzed data and wrote the manuscript. J.W., J.L., Y.D., Z.K., X.Q., X.O., K.D., Q.C., J.L., X.C., K.D., L.Z., and M.L. performed some of the experiments. Z.L., M.J., A.S., K.S., F.L., G.Z., K.W., Y.R., V.H., D.H., Y.L., K.H. and Y.L. reviewed and edited the manuscript. D.H., and S.L. conceived of and designed experiments. D.H. did the project administration. All authors approved the final manuscript.

## Supporting information

Supporting Information

## Data Availability

The data that support the findings of this study are available in the supplementary material of this article.
