# Supplementary material for: Aloperine Suppresses Cancer Progression by Interacting with VPS4A to Inhibit Autophagosome‐lysosome Fusion in NSCLC
Source: Adv Sci (Weinh). 2024 Jun 21;11(31):2308307. doi: 10.1002/advs.202308307 (PMC11336898; doi:10.1002/advs.202308307)
Supplement: Supplementary file 1 — Supporting Information [file ADVS-11-2308307-s001.docx]

Supporting Information

Aloperine Suppresses Cancer Progression by Interacting with VPS4A to Inhibit Autophagosome-lysosome Fusion in NSCLC

Weina Guo, Haifeng Zhou, Jingbo Wang, Junjie Lu, Yalan Dong, Zhenyu Kang, Xiaoyuan Qiu, Xiaohu Ouyang, Qianyun Chen, Junyi Li, Xiang Cheng, Keye Du, Mingyue Li, Zhihao Lin, Min Jin, Lei Zhang, Alexey Sarapultsev, Kuangyu Shi, Fangfei Li, Ge Zhang, Kongming Wu, Yueguang Rong, Vigo Heissmeyer, Yue Liu, Yunlun Li, Kun Huang, Shanshan Luo, Desheng Hu*

Figure S1


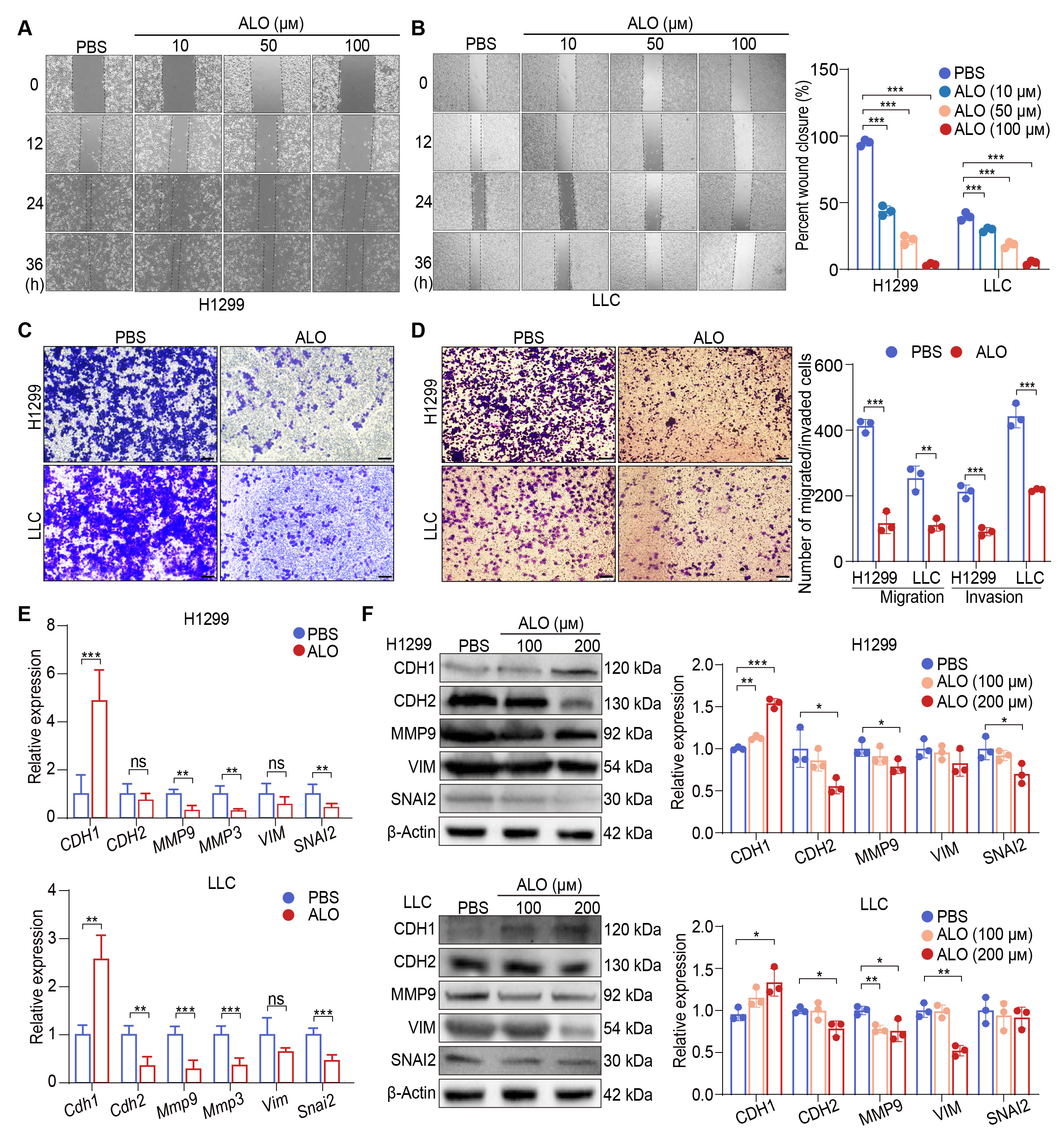


**Figure S1.** ALO inhibits cell migration of NSCLC cells. A,B) Representative results of wound-healing assays (n = 3). C) Transwell migration assay with the 24-well Transwell system (n = 3). Scale bar: 100 μm. D) Transwell invasion assay with the 24-well Transwell system (n = 3). Scale bar: 100 μm. E) The mRNA levels of several key regulators of cell metastatic signal, including cadherin-1 (CDH1), cadherin-2 (CDH2), matrix metalloproteinase-9 (MMP9), matrix metalloproteinase-3 (MMP3), vimentin (VIM), and SNAI2 in the H1299 and LLC cells were examined by RT-qPCR after treatment with ALO (200 μм) for 24 h (n = 3). F) Immunoblotting assays were performed to assess CDH1, CDH2, MMP9, VIM, and SNAI2 levels in the H1299 and LLC cells after treatment with ALO (200 μм) for 24 h and quantified by gray scale analysis (n = 3). Data in B) and F) are presented as mean ± SD, and *p* values were calculated using one‐way ANOVA. Data in D–E) are presented as mean ± SD, and *p* values were determined by two‐tailed unpaired Student’s t-test. ns, not significant; **p* < 0.05, ***p* < 0.01, ****p* < 0.001.

Figure S2


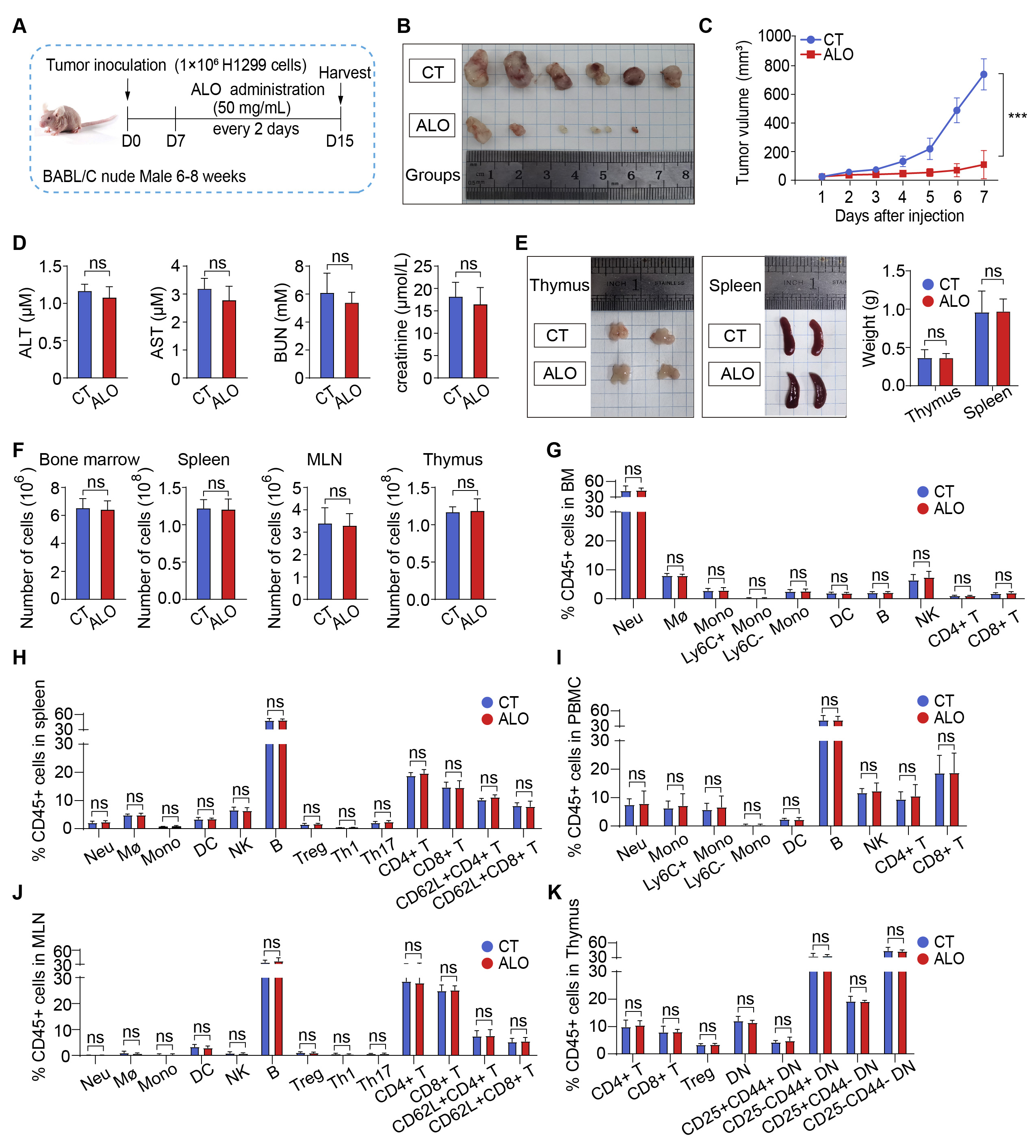


**Figure S2.** ALO exerts antitumor efficacy in H1299-derived subcutaneous tumor mouse models while exhibiting no significant side effects on the general state of mice. A) BALB/c nude mice bearing H1299-derived subcutaneous tumors were randomly divided into two groups: the control group (CT) and the ALO group (ALO), receiving PBS or ALO (50 mg kg^−1^) every 2 days, respectively (n = 6). B) Images of H1299-derived subcutaneous tumors. C) Tumor growth curves illustrate the significant inhibitory effect of ALO on the growth of H1299-derived subcutaneous tumors (n = 6). D) Comparison of serum levels of alanine transaminase (ALT), aspartate aminotransferase (AST), blood urea nitrogen (BUN) and creatinine in ALO-treated mice versus the control group (CT) (n = 5). E) Images and weights of thymuses and spleens collected from the C57BL/6J mice of the CT and 50 mg kg^−1^ ALO-treated groups (n = 5). F) Total cell counts in bone marrows (BMs), spleens, mesenteric lymph nodes (MLNs), and thymuses collected from the mice of the CT and ALO-treated groups (n = 5). G) Percentage of each cell population in the BMs of ALO-treated mice compared with the CT group (n = 5). H) Percentage of each cell population in the spleens of ALO-treated mice compared with the CT group (n = 5). I) Percentage of each cell population in the peripheral blood mononuclear cells (PBMCs) of ALO-treated mice compared with the CT group (n = 5). J) Percentage of each cell population in the MLNs of ALO-treated mice compared with the CT group (n = 5). K) Percentage of each cell population in the thymuses of ALO-treated mice compared with the CT group (n = 5). Data in A–K) are presented as mean ± SD, and *p* values were determined by two‐tailed unpaired Student’s t-test. ns, not significant; **p* < 0.05, ***p* < 0.01, ****p* < 0.001. Neu, neutrophils. Mø, macrophages. Mono, monocytes. DC, dentric cells. B, B cells. NK, natural killer cells. Treg, regulatory T cells. Th1, T helper 1 cells.

Figure S3


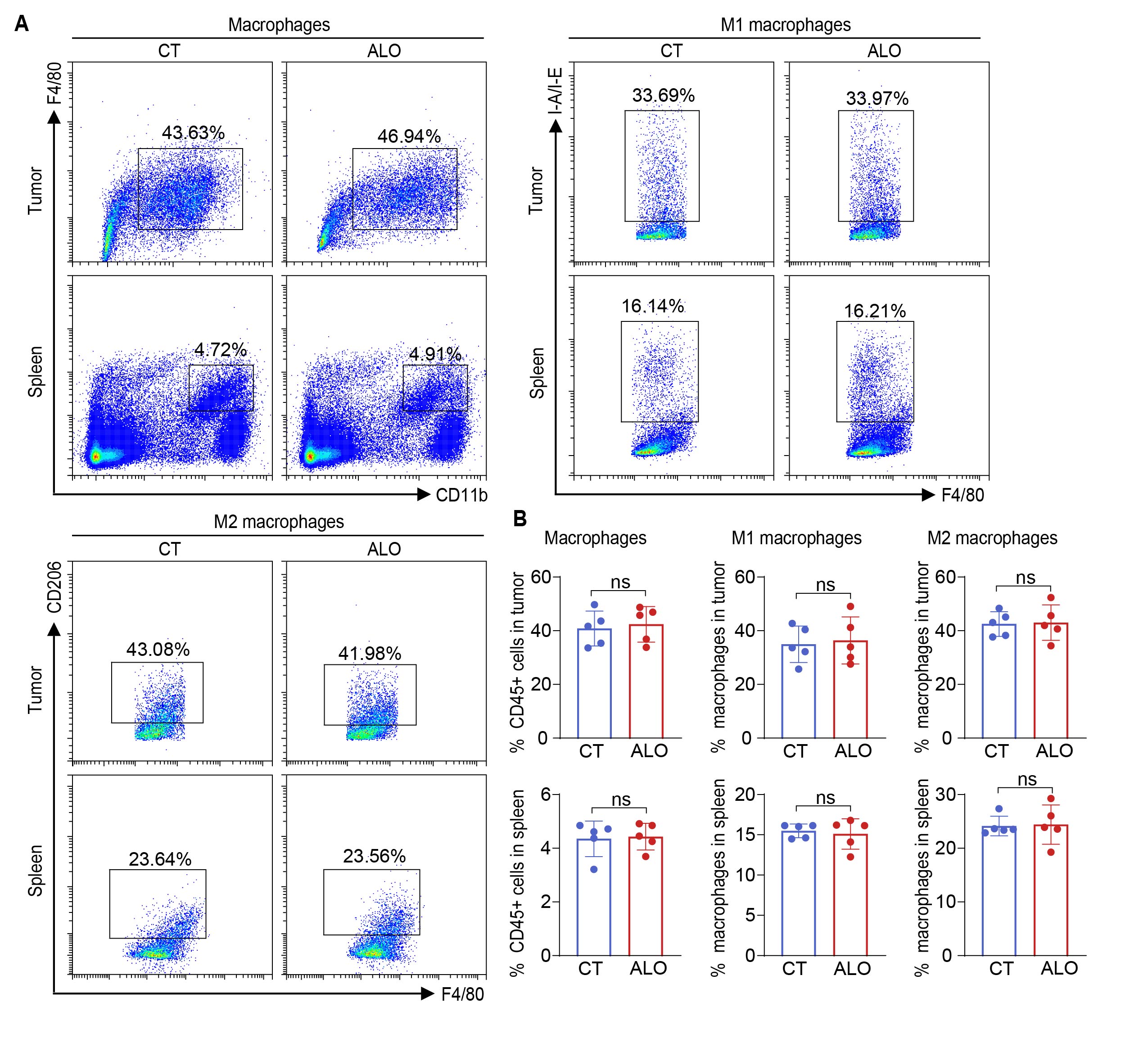


**Figure S3.** ALO does not affect the proportion or polarization of macrophages. A) Analysis of total macrophages, M1 macrophages and M2 macrophages in tumors and spleens of LLC-derived subcutaneous tumor mouse models from each group was conducted by flow cytometry (n = 5). B) Analysis of the proportion of total macrophages, M1 macrophages and M2 macrophages in tumors and spleens of LLC-derived subcutaneous tumor mouse models from each group (n = 5). Data are presented as mean ± SD, and *p* values were determined by two-tailed unpaired Student’s t-test. ns, not significant.

Figure S4


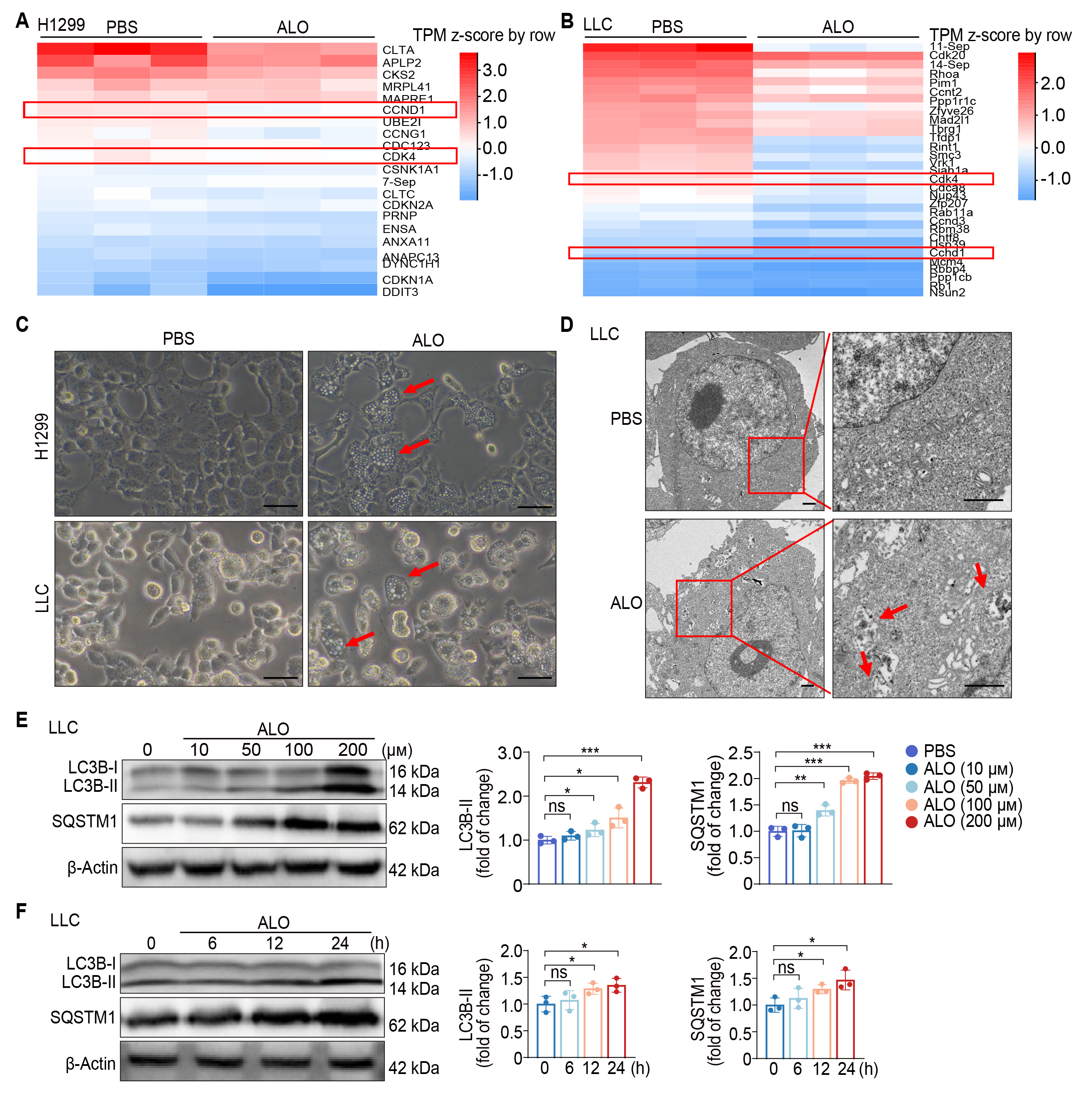


**Figure S4.** ALO modulates autophagy in NSCLC cells. A,B) Heatmaps of the differentially expressed genes related to cell cycle pathway in ALO-treated and untreated H1299 A) and LLC cells B). C) Microscopy images of H1299 and LLC cells treated with the PBS or ALO (200 μм) for 12 h. Scale bar: 50 μm. D) Transmission electron micrographs of LLC cells treated with PBS or ALO (200 μм) for 24 h. The right images are the enlarged representations of the boxed regions of the left images. Scale bar: 1 μm. E) Immunoblotting assays were performed to assess LC3B-II and SQSTM1 levels in LLC cells treated with ALO (0–200 μм) for 24 h and quantified by gray scale analysis (n = 3). F) Immunoblotting assays were performed to assess LC3B-II and SQSTM1 levels in LLC cells treated with ALO (200 μм) for indicated time periods and quantified by gray scale analysis (n = 3). β-Actin was used as a loading control. Data in E,F) are presented as mean ± SD, and *p* values were calculated using one‐way ANOVA. ns, not significant; **p* < 0.05, ***p* < 0.01, ****p* < 0.001.

Figure S5


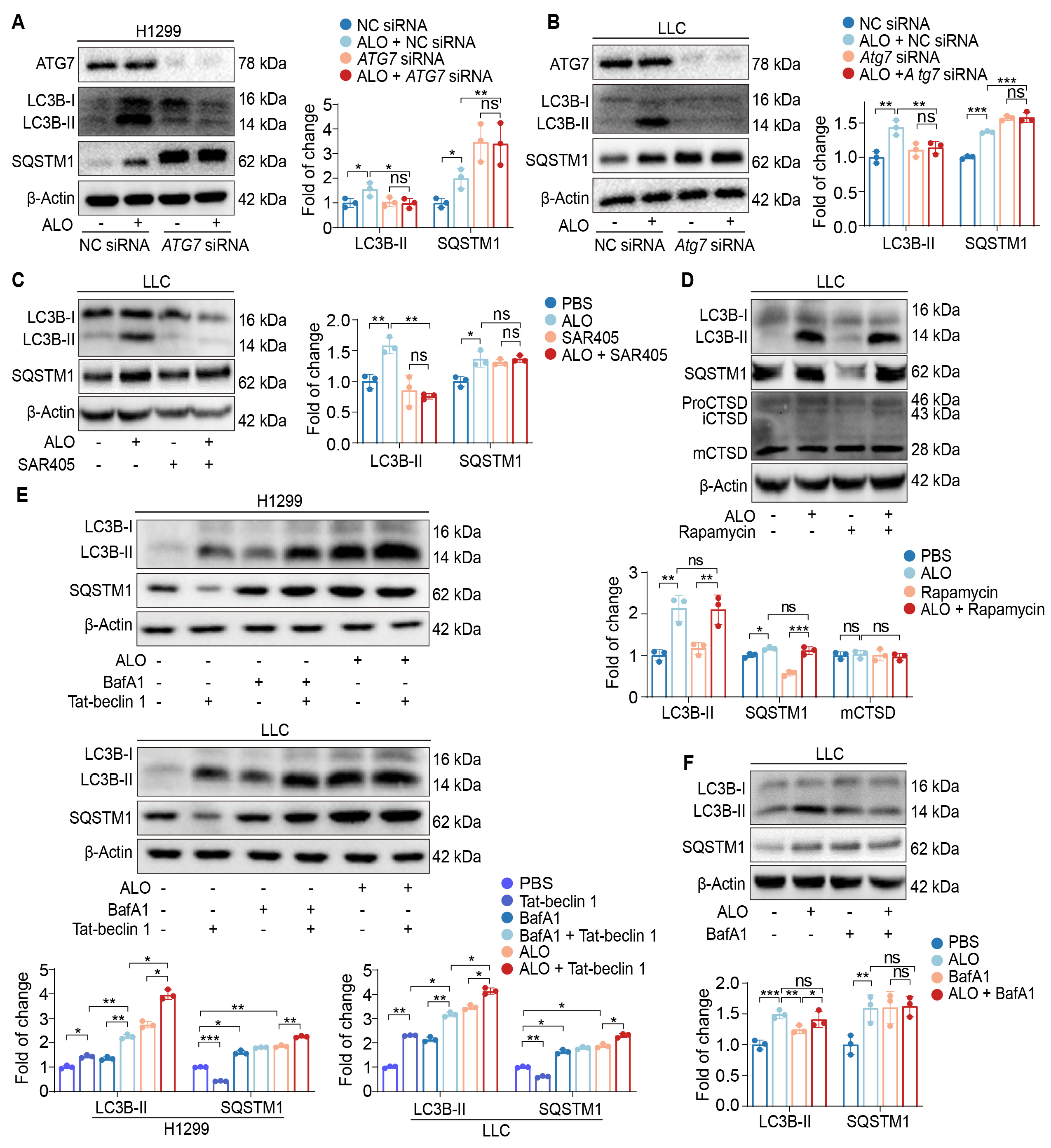


**Figure S5.** ALO might promote autophagosome formation and impair autophagosome degradation. A) Immunoblotting assays were performed to assess ATG7, LC3B-II and SQSTM1 levels in H1299 cells treated with PBS or ALO (200 μм) after transfection with *ATG7* siRNA or negative control (NC) siRNA and quantified by gray scale analysis (n = 3). B) Immunoblotting assays were performed to assess ATG7, LC3B-II and SQSTM1 levels in LLC cells treated with PBS or ALO (200 μм) after transfection with *Atg7* siRNA or NC siRNA, and quantified by gray scale analysis (n = 3). C) Immunoblotting assays were performed to assess LC3B-II and SQSTM1 levels in LLC cells treated with PBS or ALO (200 μм) in the absence or presence of 10 μм SAR405 for 2 h and quantified by gray scale analysis (n = 3). D) Immunoblotting assays were performed to assess LC3B-II, SQSTM1, procathepsin D (pro-CTSD), preprocathepsin D (pre-CTSD) and mature CTSD (mCTSD) levels in LLC cells treated with PBS or ALO (200 μм) in the absence or presence of rapamycin (200 nм, 12 h) for 2 h and quantified by gray scale analysis (n = 3). E) Immunoblotting assays were performed to assess LC3B-II and SQSTM1 levels in H1299 and LLC cells treated with PBS or ALO (200 μм) in the absence or presence of 40 μм Tat-beclin 1 or 1 μм Bafilomycin A1 (BafA1) for 2 h and quantified by gray scale analysis (n = 3). F) Immunoblotting assays were performed to assess LC3B-II and SQSTM1 levels in LLC cells treated with PBS or ALO (200 μм) in the absence or presence of 1 μм BafA1 for 2 h and quantified by gray scale analysis (n = 3). Data in A–F) are presented as mean ± SD, and *p* values were calculated using one‐way ANOVA. ns, not significant; **p* < 0.05, ***p* < 0.01, ****p* < 0.001.

Figure S6


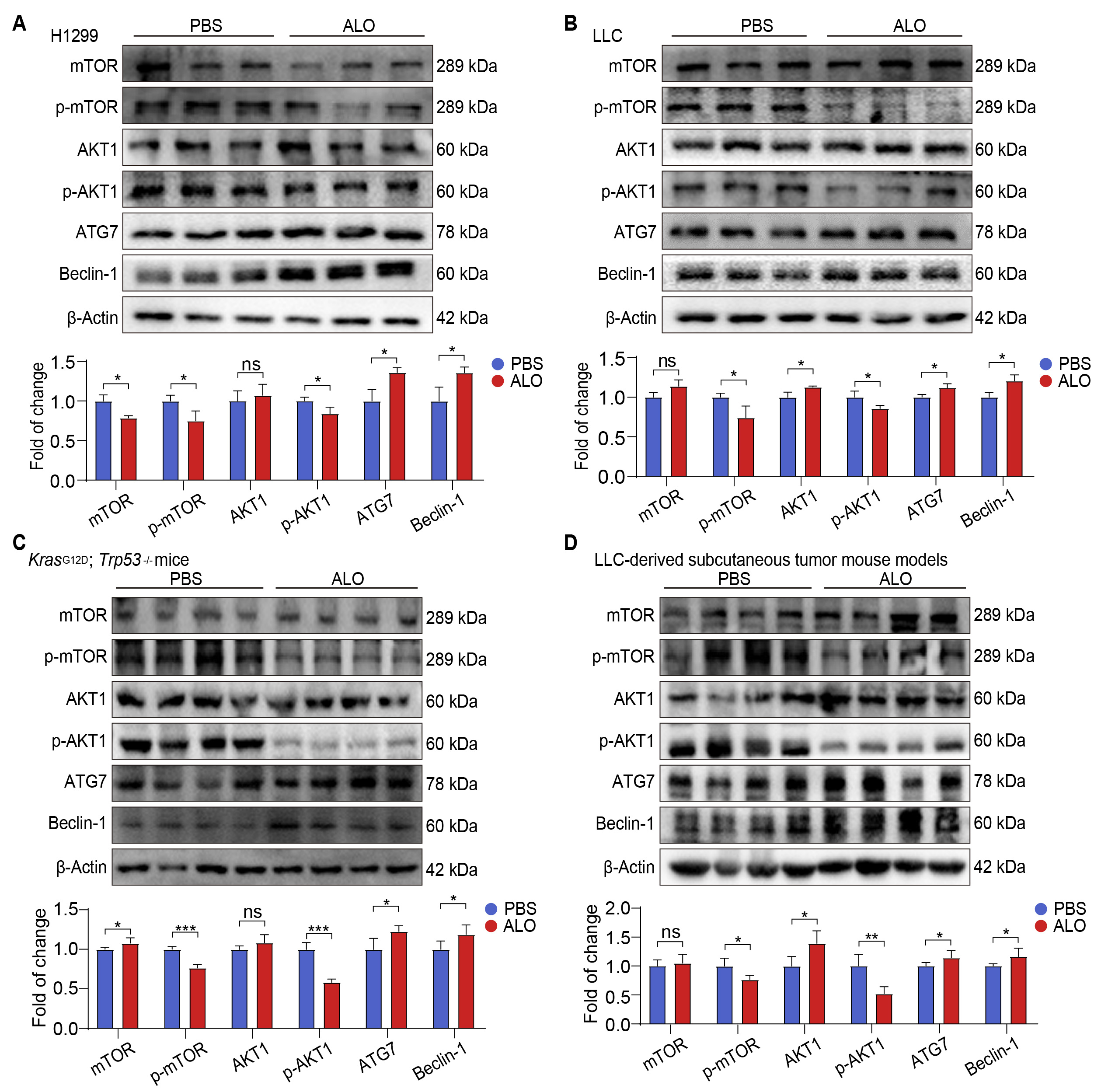


**Figure S6.** ALO might promote autophagosome formation by suppressing the Akt/mTOR pathway. A,B) Immunoblotting assays were performed to assess mTOR, p-mTOR, AKT1, p-AKT1, ATG7 and Beclin-1 levels in H1299 A) and LLC cells B) treated with PBS or ALO (200 μм) for 24 h and quantified by gray scale analysis (n = 3). C,D) Immunoblotting assays were performed to assess mTOR, p-mTOR, AKT1, p-AKT1, ATG7 and Beclin-1 levels in tumor tissues collected from *Kras*^G12D^;*Trp53*^-/-^ mice C) (n = 4) and LLC-derived subcutaneous tumor mouse models with ALO treatment D) (n = 4). Data in A–D) are presented as mean ± SD, and *p* values were determined by two‐tailed unpaired Student’s t-test. ns, not significant; **p* < 0.05, ***p* < 0.01, ****p* < 0.001.

Figure S7


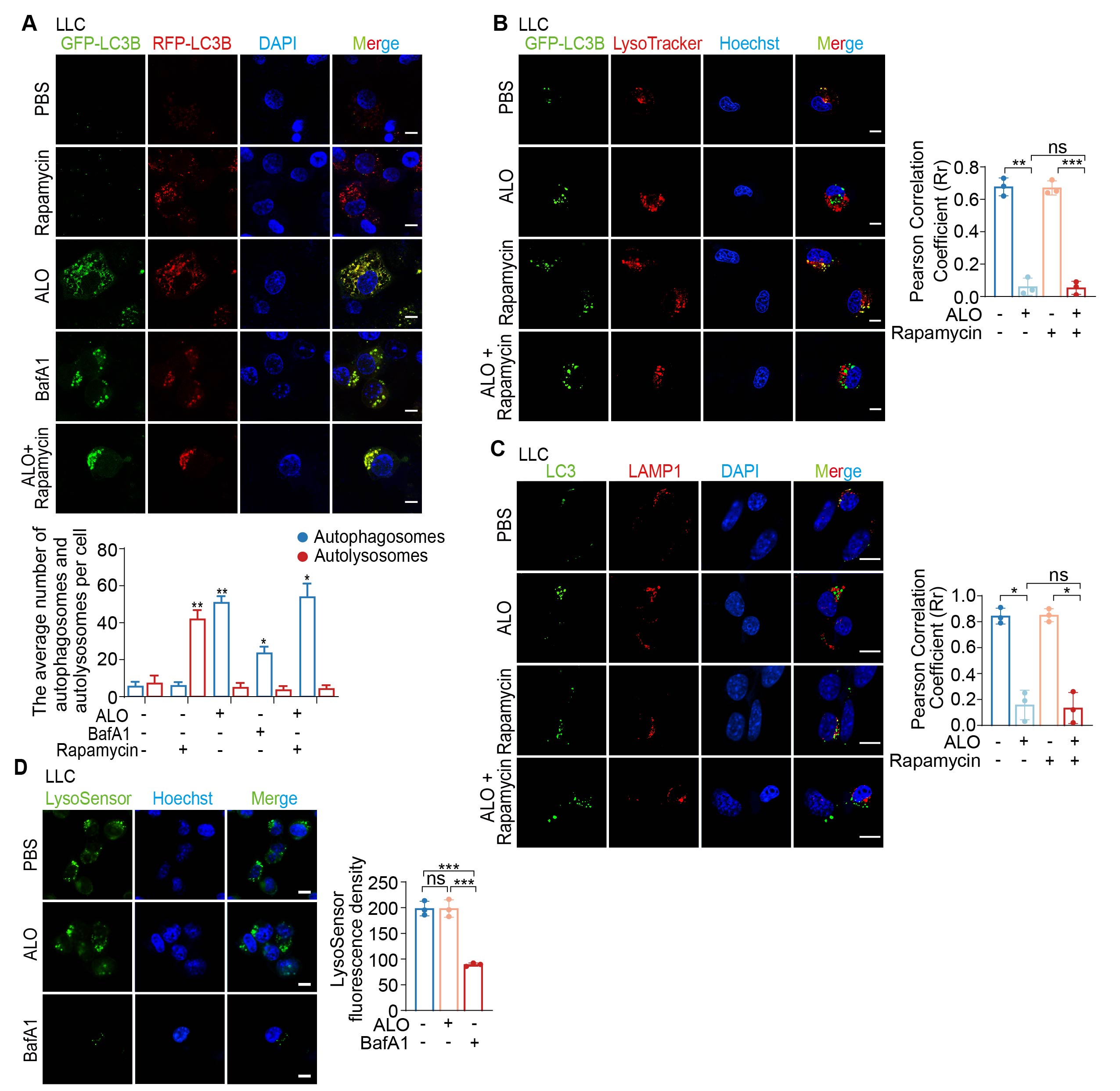


**Figure S7**. ALO inhibits autophagic flux by blocking autophagosome-lysosome fusion. A) Fluorescence images of LLC cells transfected with RFP-GFP-LC3B reporter. Cells were treated with PBS or ALO (200 μм) in complete medium for 2 h. 1 μм Bafilomycin A1 (BafA1)-treated cells served as positive controls (n = 3). Scale bar: 10 μm. B) Fluorescence images of the colocalization of GFP-LC3B and LysoTracker Red in LLC cells cultured in full medium in the absence or presence of ALO (200 μм) for 2 h (n = 3). Scale bar: 10 μm. C) Immunofluorescence images and analysis of the colocalization of LC3 (green) and LAMP1 (red) in LLC cells treated with PBS or ALO (200 μм) for 2 h (n = 3). Scale bar: 10 μm. D) LLC cells were treated with PBS or ALO (200 μм) for 2 h. Representative images of LLC cells stained with LysoSensor Green and quantification of fluorescence intensity (n = 3). 1 μм BafA1-treated cells served as positive controls. Scale bar: 10 μm. Data in A–D) are presented as mean ± SD, and *p* values were calculated using one‐way ANOVA. ns, not significant; **p* < 0.05; ***p* < 0.01; ****p* < 0.001.

Figure S8


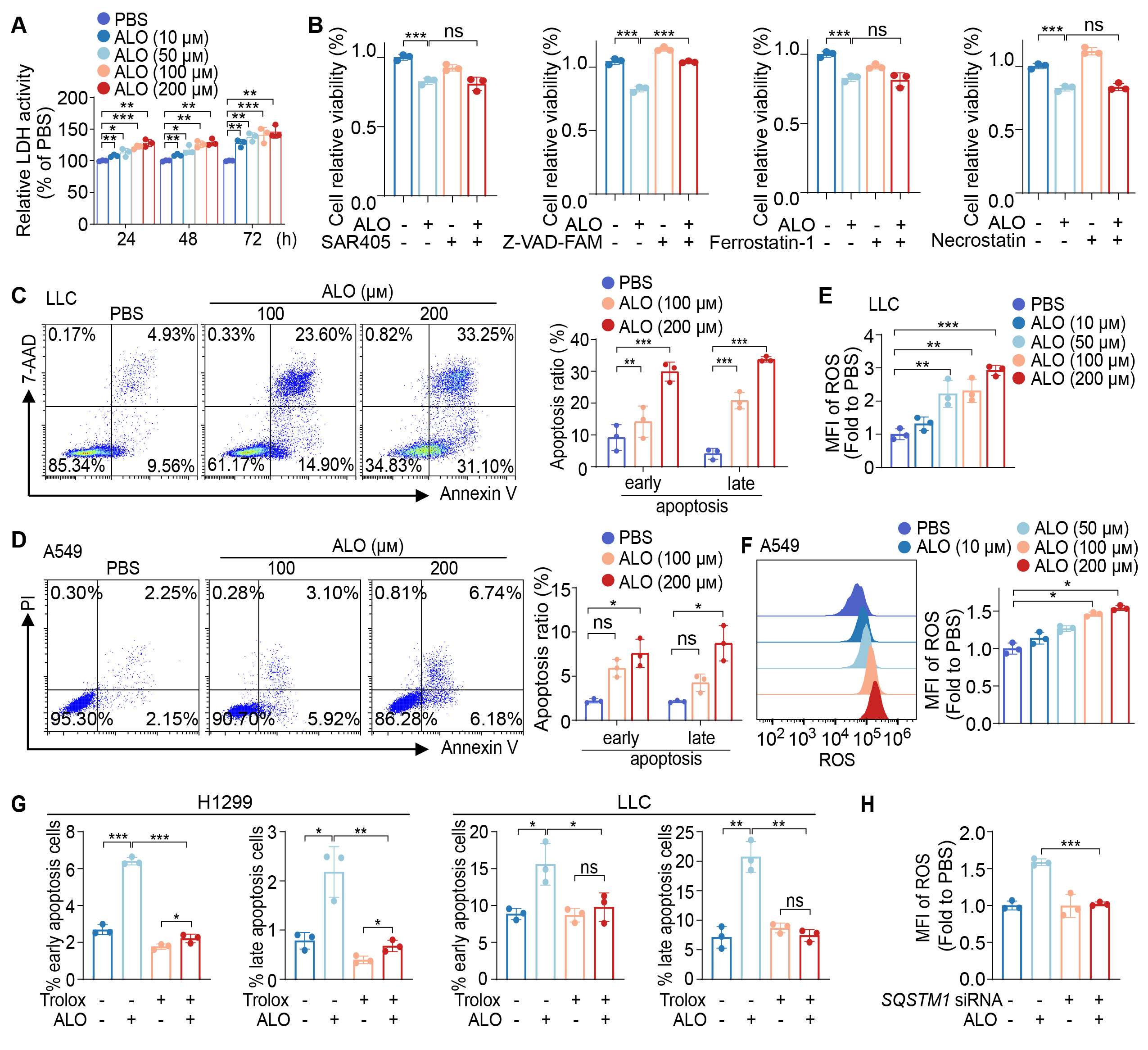


**Figure S8.** ALO-mediated cell apoptosis is mediated by excessive reactive oxygen species (ROS) production. A) The cytotoxicity of ALO on LLC cells was measured using the LDH releasing assay (n = 3). B) Modulatory profiling of known small-molecule cell death inhibitors in LLC cells treated with ALO (200 μм, 24 h) (n = 3). C) Representative results of annexin V/7-AAD staining in LLC cells treated with ALO (0–200 μм) for 48 h (n = 3). D) Representative results of Annexin V/PI staining in A549 cells treated with ALO (0–200 μм) for 48 h (n = 3). E) The intracellular level of ROS was measured in LLC cells treated with ALO (200 μм) for 24 h (n = 3). F) The ROS level was measured in A547 cells treated with ALO (200 μм) for 24 h (n = 3). G) Annexin V/7-AAD staining was performed to estimate the ratio of cellular apoptosis in ALO-treated H1299 and LLC cells in the absence or presence of Trolox (n = 3). H) The intracellular ROS level induced by ALO in H1299 transfected with *SQSTM1* siRNA or negative control siRNA (n = 3). Data in A–H) are presented as mean ± SD, and *p* values were calculated using one‐way ANOVA. ns, not significant; **p* < 0.05, ***p* < 0.01, ****p* < 0.001.

Figure S9


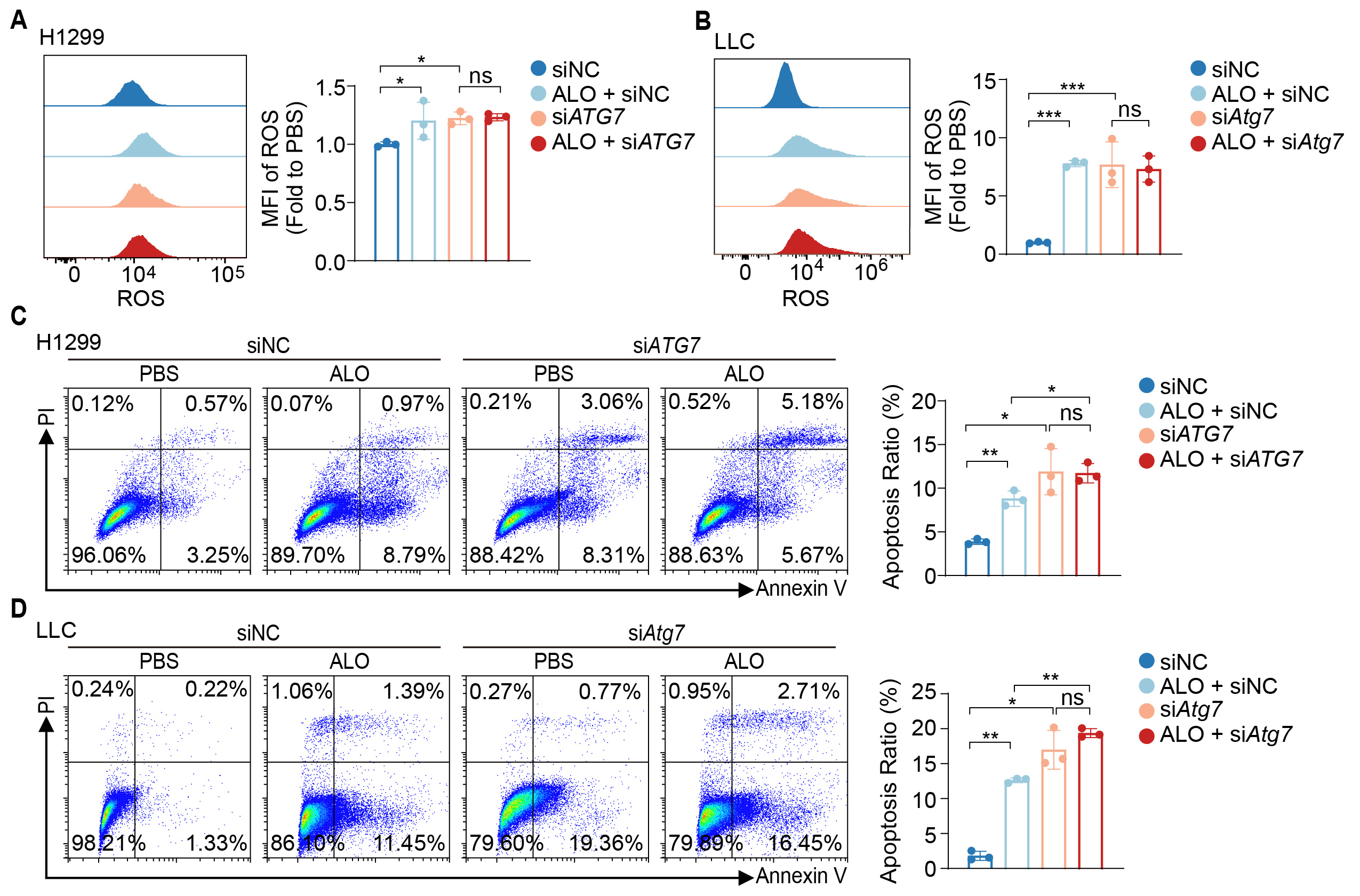


**Figure S9.** ALO-induced reactive oxygen species (ROS) accumulation and apoptosis in NSCLC cells are mediated by autophagy inhibition. A) The intracellular level of ROS was measured in H1299 cells transfected with ATG7 siRNA (si*ATG7*) or negative control siRNA (siNC) after treatment of ALO (200 μм) for 24 h (n = 3). B) The intracellular ROS level was measured in LLC cells transfected with *Atg7* siRNA (si*Atg7*) or siNC after treatment of ALO (200 μм) for 24 h (n = 3). C) Annexin V/PI staining was performed to estimate the ratio of cellular apoptosis in ALO-treated H1299 cells transfected with si*ATG7* or siNC (n = 3). D) Annexin V/PI staining was performed to estimate the ratio of cellular apoptosis in ALO-treated LLC cells transfected with si*Atg7* or siNC (n = 3). Data in A–D) are presented as mean ± SD, and *p* values were calculated using one‐way ANOVA. ns, not significant; **p* < 0.05, ***p* < 0.01, ****p* < 0.001.

Figure S10


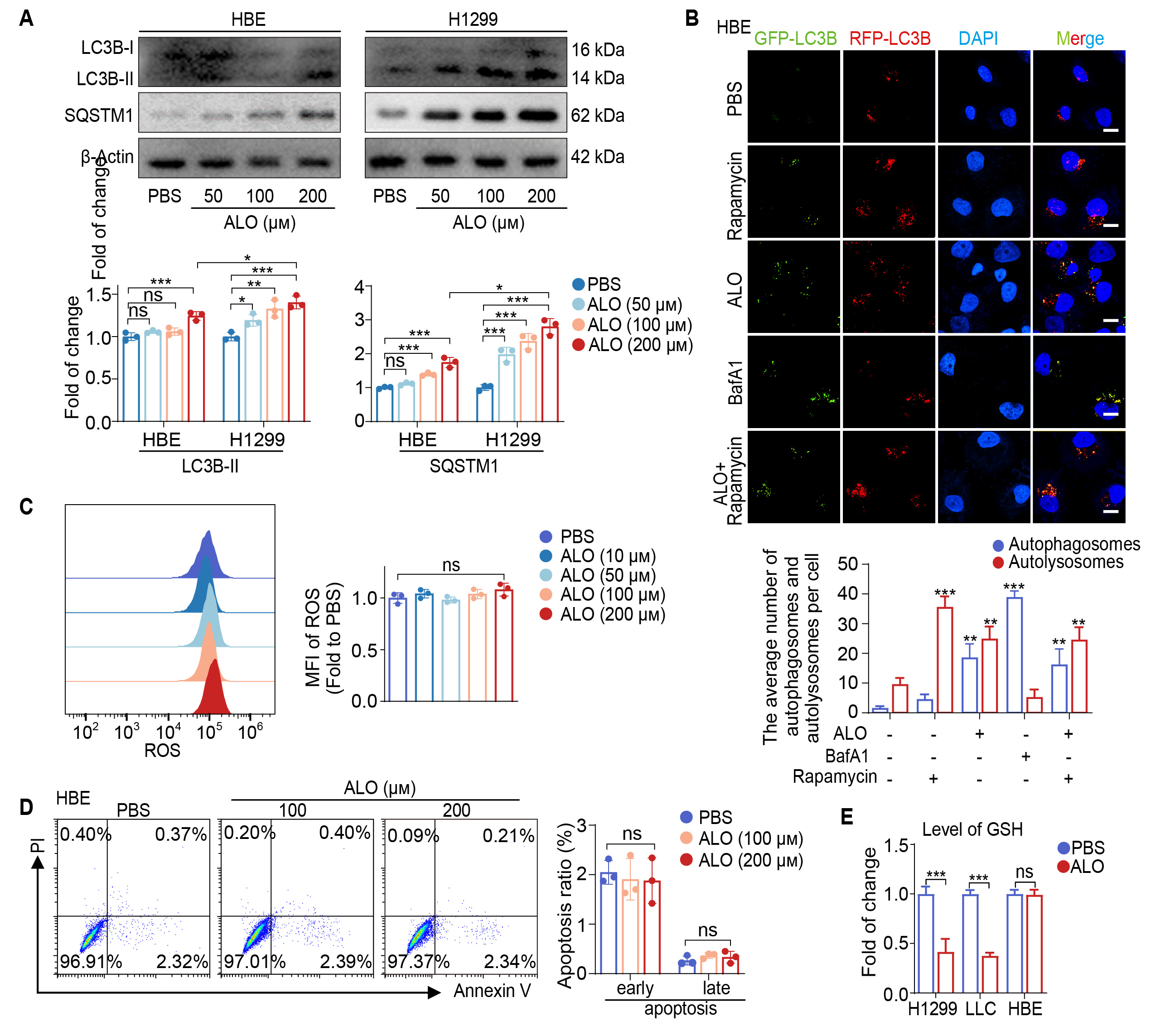


**Figure S10.** ALO modulates autophagic flux without inducing apoptosis in HBE cells. A) Immunoblotting assays were performed to assess LC3B-II and SQSTM1 levels in HBE and H1299 cells treated with PBS or ALO (0–200 μм) and quantified by gray scale analysis (n = 3). B) Fluorescence images of HBE cells transfected with RFP-GFP-LC3B reporter and analysis of the number of autophagosomes and autolysosomes. Cells were treated with PBS or ALO (200 μм) in complete medium for 2 h. 1 μм Bafilomycin A1 (BafA1)-treated cells were used as positive controls (n = 3). Scale bar: 10 μm. C) The intracellular level of reactive oxygen species (ROS) was measured in HBE cells treated with ALO (200 μм) for 24 h (n = 3). D) Flow cytometry analysis of Annexin V/PI staining in HBE cells treated with ALO (0–200 μм) for 48 h (n = 3). E) Total glutathione (GSH) content in HBE, H1299 and LLC cells were determined by using a GSH assay kit after incubation with ALO (200 μм) for 24 h. Data in A–D) are presented as mean ± SD, and *p* values were calculated using one‐way ANOVA. Data in E) are presented as mean ± SD, and *p* values were determined by two‐tailed unpaired Student’s t-test. ns, not significant; **p* < 0.05, ***p* < 0.01, ****p* < 0.001.

Figure S11


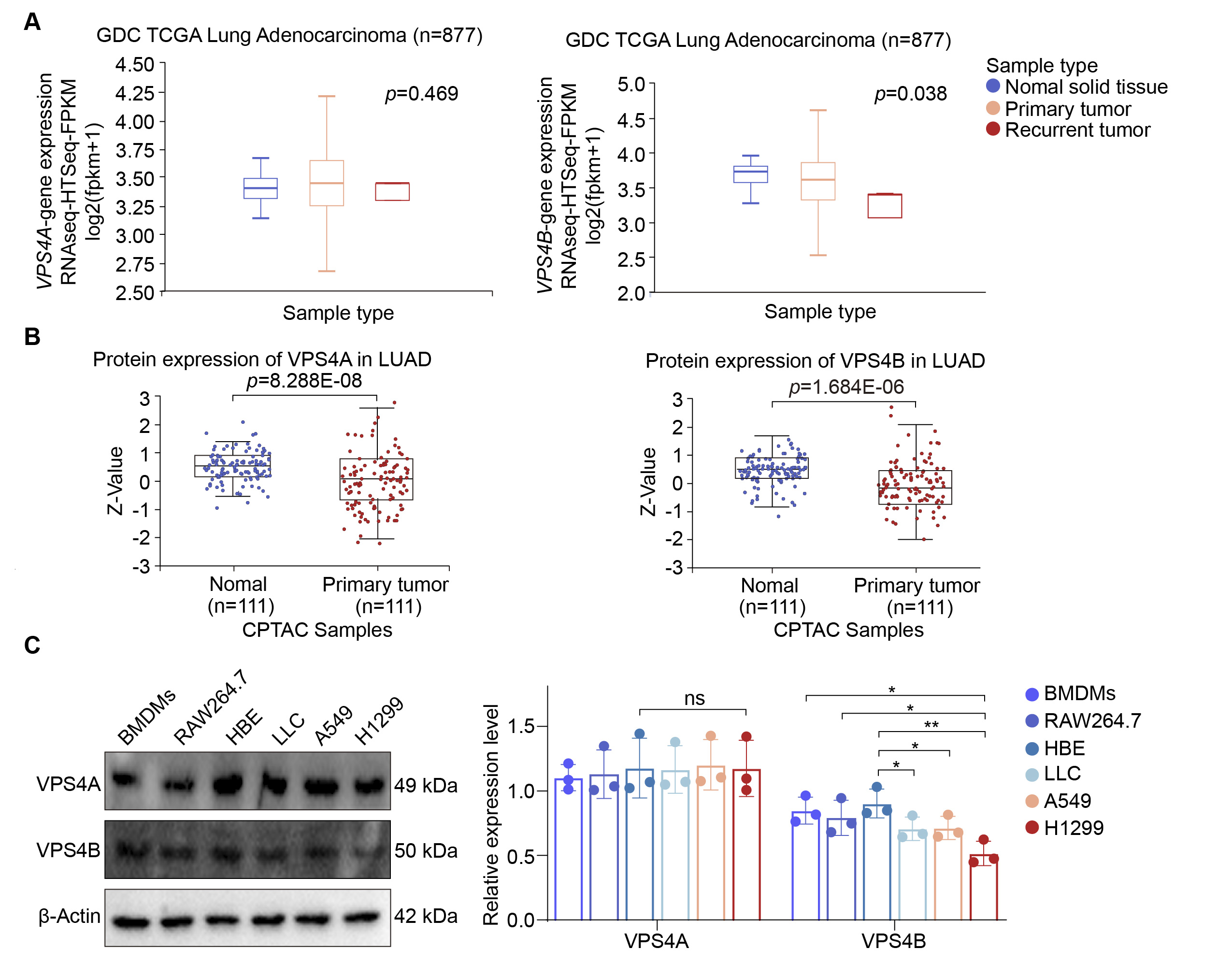


**Figure S11.** Expression levels of VPS4A and VPS4B in lung adenocarcinoma (LUAD) patient samples and various cell lines. A) mRNA expression analysis of *VPS4A* and *VPS4B* was conducted using data from the Cancer Genome Atlas (TCGA) LUAD patient samples (n = 877) via the UCSC Xena browser. The *p* values were calculated using one‐way ANOVA. B) Protein expression analysis of VPS4A and VPS4B in normal and primary tumors (n = 111) from the LUAD datasets of Clinical Proteomic Tumor Analysis Consortium (CPTAC) was performed using the UALCAN portal. Z-values represent standard deviations from the median across samples for LUAD. The *p* values were determined by two‐tailed unpaired Student’s t-test. C) Immunoblotting assays were performed to assess VPS4A and VPS4B levels in various cell lines, including bone marrow-derived macrophages (BMDMs), RAW264.7, human bronchial epithelial cells (HBE), LLC, A549, H1299, and quantified by gray scale analysis (n = 3). Data are presented as mean ± SD, and *p* values were calculated using one‐way ANOVA. ns, not significant; **p* < 0.05, ***p* < 0.01, ****p* < 0.001.

Figure S12


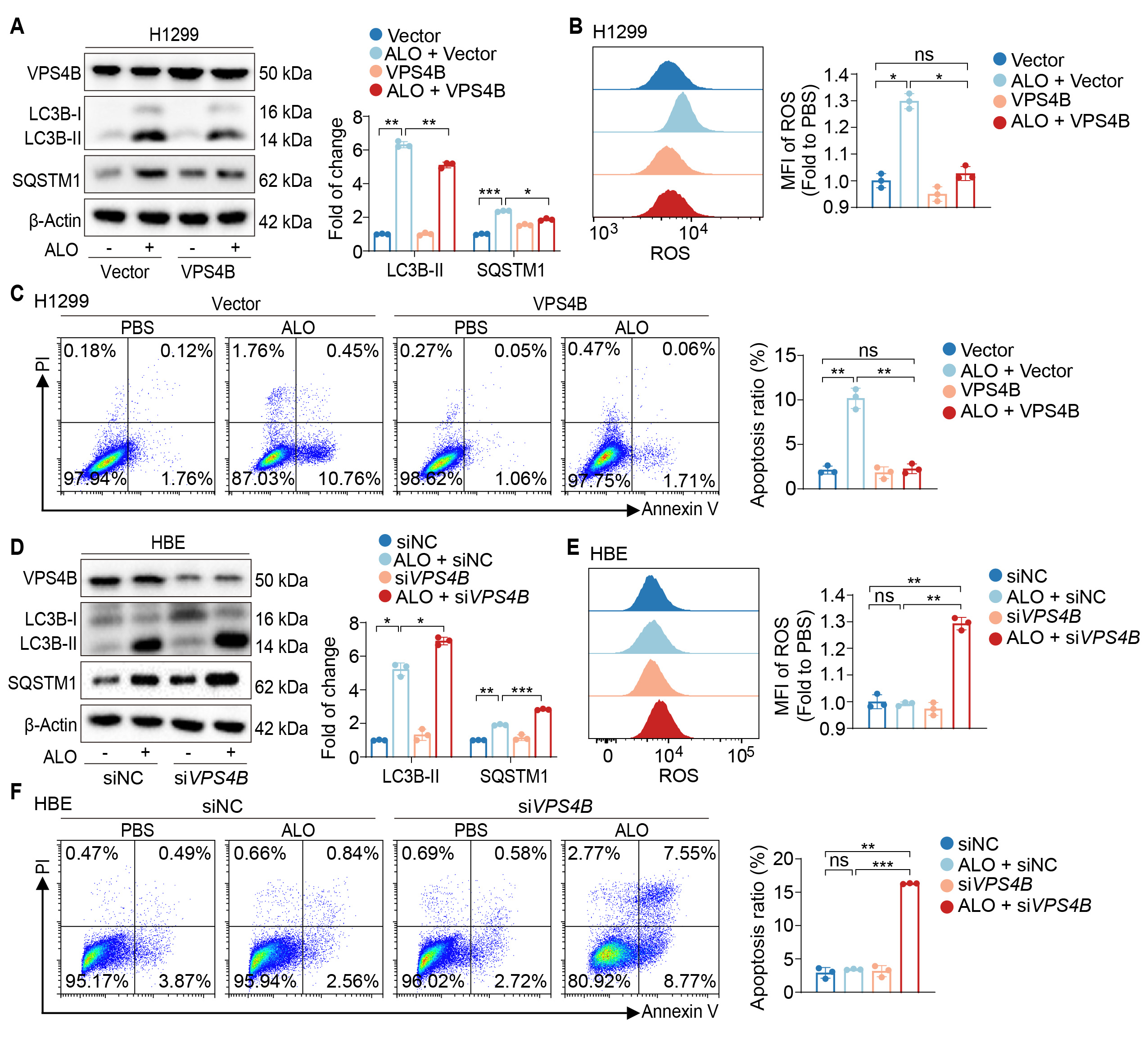


**Figure 12.** The imbalance between VPS4A and VPS4B affects the sensitivity of cells to ALO. A) Immunoblotting assays were performed to assess VPS4B, LC3B-II and SQSTM1 levels in H1299 cells treated with PBS or ALO (200 μм) after transfection with plasmids overexpressing VPS4B or vector, and quantified by gray scale analysis (n = 3). B) The intracellular level of reactive oxygen species (ROS) was measured in H1299 cells transfected with the plasmids overexpressing VPS4B or vector after treatment of ALO (200 μм) for 24 h (n = 3). C) Annexin V/PI staining was performed to estimate the ratio of cellular apoptosis in ALO-treated H1299 cells transfected with the plasmids overexpressing VPS4B or vector (n = 3). D) Immunoblotting assays were performed to assess VPS4B, LC3B-II and SQSTM1 levels in HBE cells treated with PBS or ALO (200 μм) after transfection with *VPS4B* siRNA (si*VPS4B*) or siNC, and quantified by gray scale analysis (n = 3). E) The intracellular ROS level was measured in HBE cells transfected with si*VPS4B* or siNC after treatment of ALO (200 μм) for 24 h (n = 3). F) Annexin V/PI staining was performed to estimate the ratio of cellular apoptosis in ALO-treated HBE cells transfected with si*VPS4B* or siNC (n = 3). Data in A–F) are presented as mean ± SD, and *p* values were calculated using one‐way ANOVA. ns, not significant; **p* < 0.05, ***p* < 0.01, ****p* < 0.001.

Figure S13


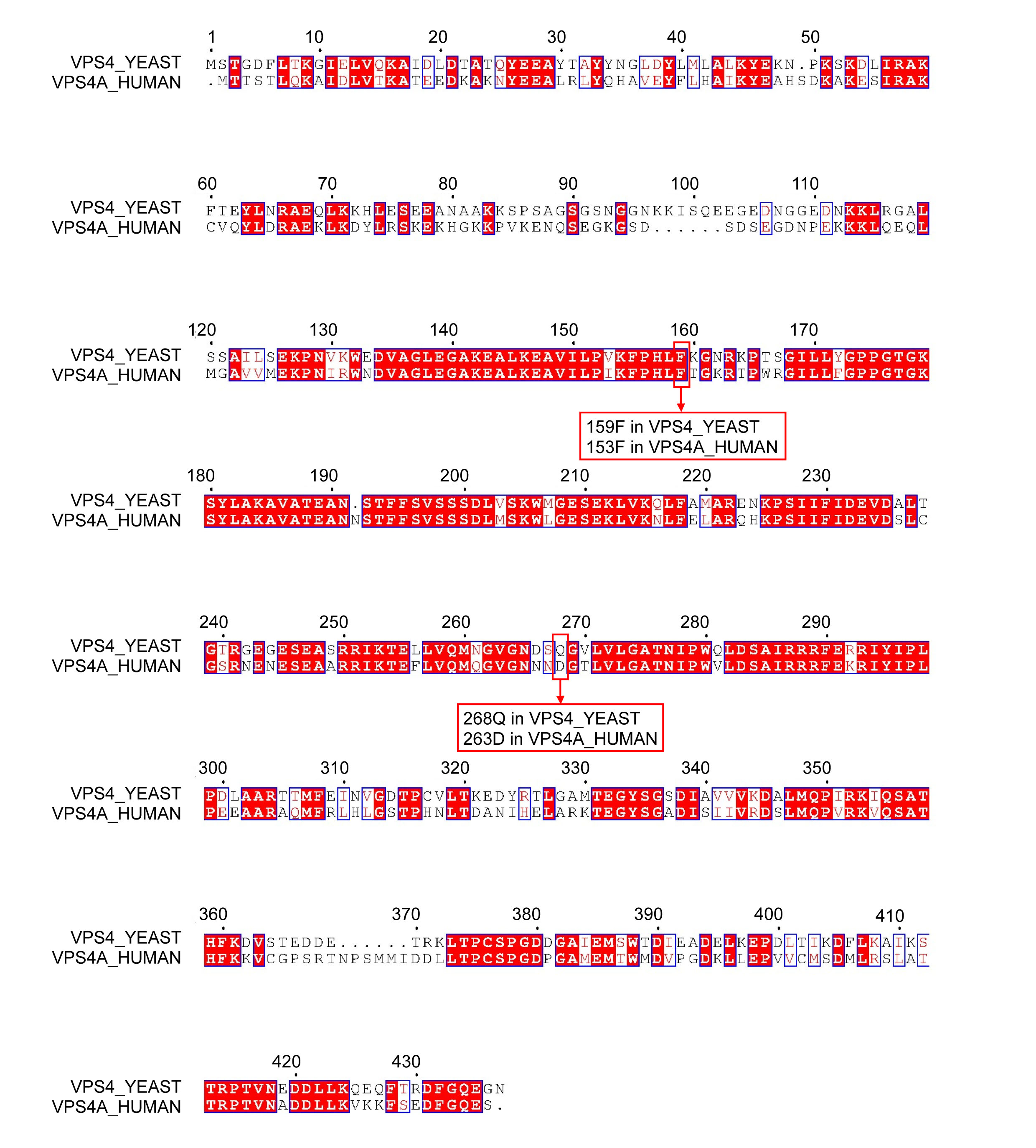


**Figure S13.** Protein sequence alignment of yeast VPS4 and human VPS4A was conducted by ESPript 3.0. The red boxes indicate amino acid residues predicted from the docking simulation to be involved in the binding of ALO within yeast VPS4.

Figure S14


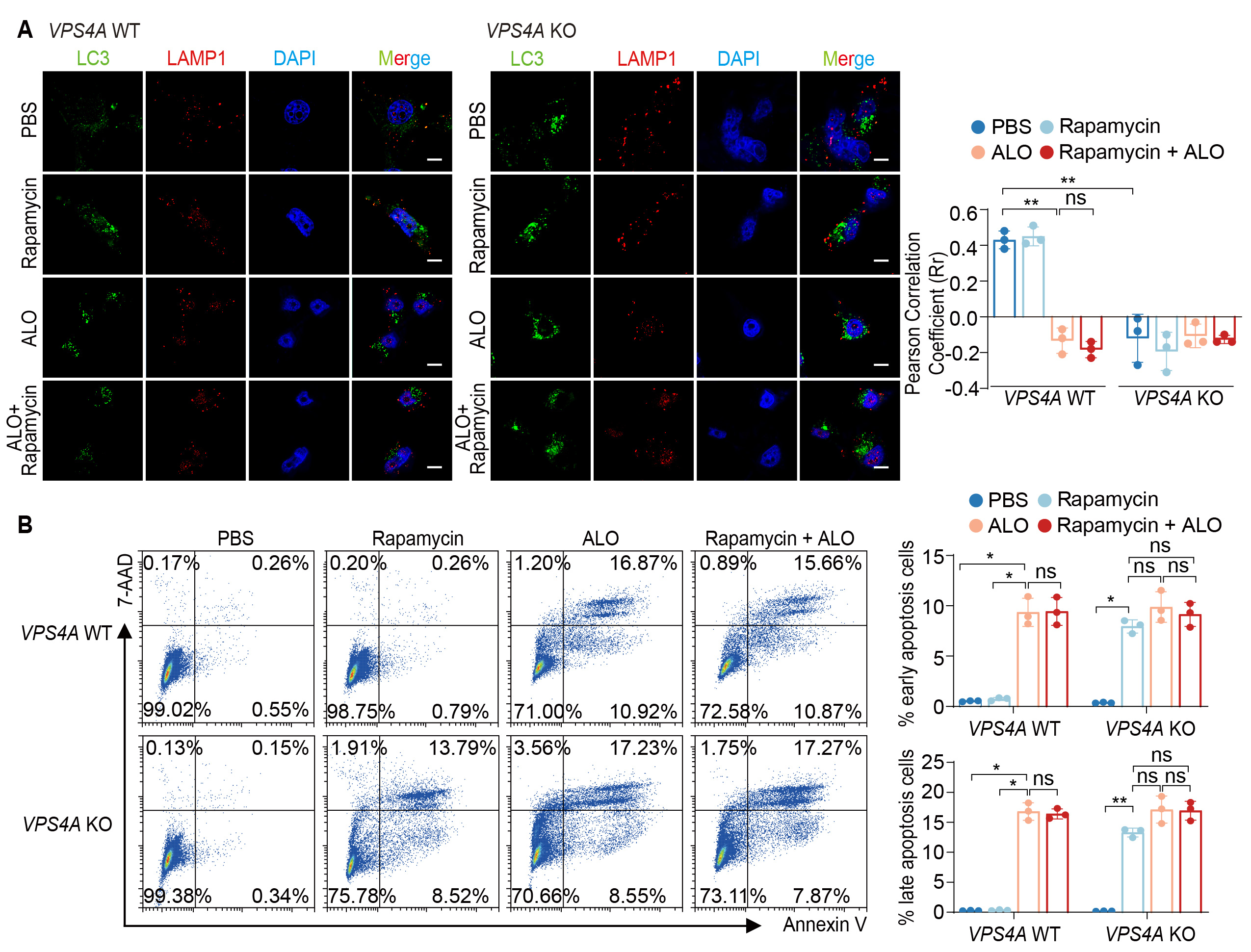


**Figure S14**. Knocking out VPS4A inhibited the fusion of autophagosomes and lysosomes. A) Fluorescence images of the colocalization of LC3 (green) and LAMP1 (red) in ALO-treated (200 μм, 2h) wild type (WT) H1299 and *VPS4A* knockout (KO) H1299 cells cultured in complete medium in the absence or presence of 250 nM rapamycin (n = 3). Scale bar: 10 μm. B) Annexin V/7-AAD staining was performed to estimate the frequencies of early and late apoptosis cells in ALO-treated WT H1299 and *VPS4A* KO H1299 cells in the absence or presence of 250 nM rapamycin (n = 3). Data in A,B) are presented as mean ± SD, and *p* values were calculated using one‐way ANOVA. ns, not significant; **p* < 0.05, ***p* < 0.01, ****p* < 0.001.

Figure S15


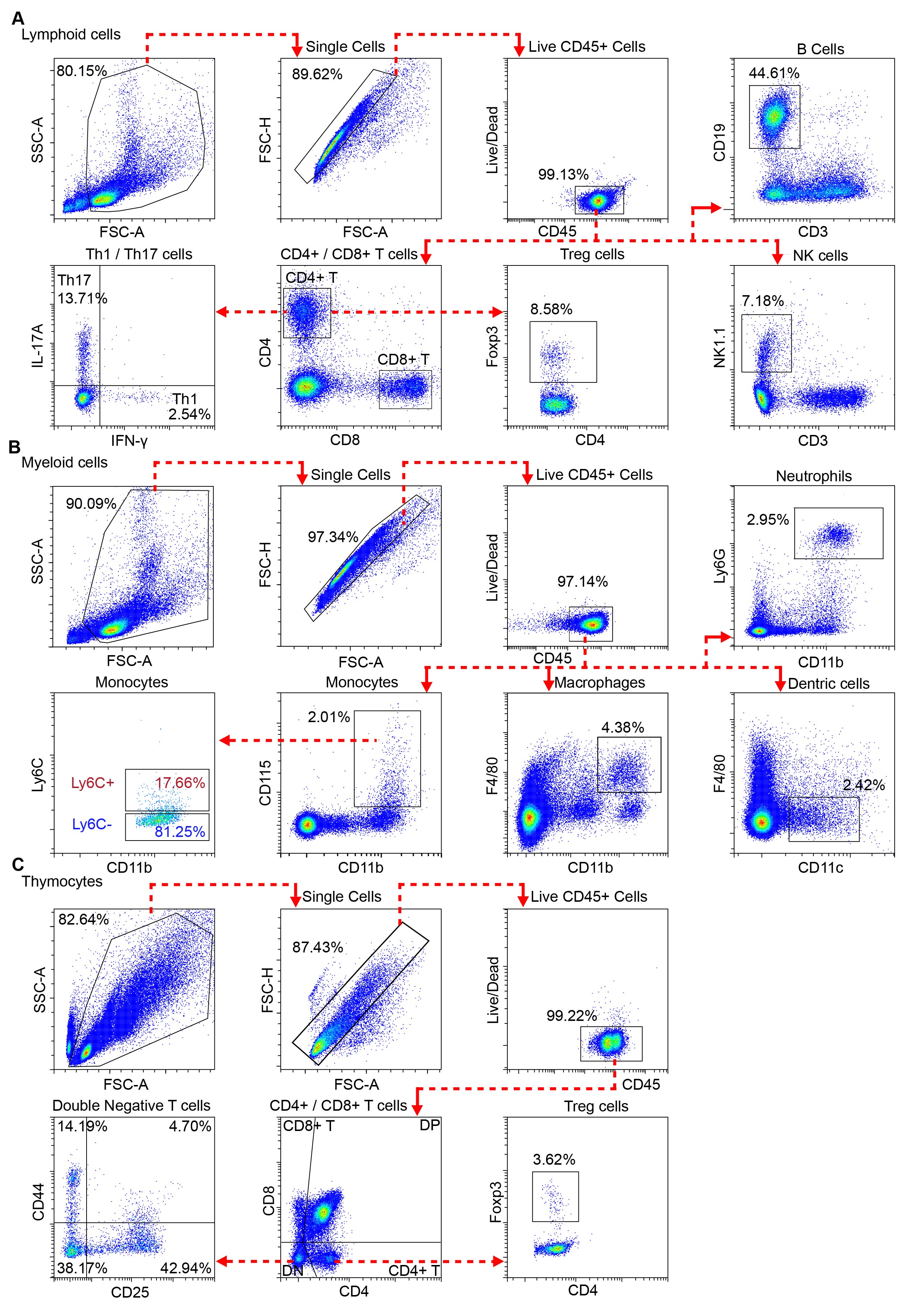


**Figure S15.** Flow gating strategies for each cell population. A) Flow gating strategies for lymphoid cells. B) Flow gating strategies for myeloid cells. C) Flow gating strategies for thymocytes. SSC-A, side scatter-area. FSC-A, forward scatter-area. FSC-H, forward scatter-height. DN, double negative. DP, double positive.
